# Rapid detection of identity-by-descent tracts for mega-scale datasets

**DOI:** 10.1038/s41467-021-22910-w

**Published:** 2021-06-10

**Authors:** Ruhollah Shemirani, Gillian M. Belbin, Christy L. Avery, Eimear E. Kenny, Christopher R. Gignoux, José Luis Ambite

**Affiliations:** 1 Information Sciences Institute, University of Southern California, Marina del Rey, CA USA; 2 Computer Science Department, University of Southern California, Los Angeles, CA USA; 3 Center for Genomic Health, Icahn School of Medicine at Mount Sinai, New York, NY USA; 4 The Charles Bronfman Institute of Personalized Medicine, Icahn School of Medicine at Mount Sinai, New York, NY USA; 5 Department of Epidemiology, University of North Carolina at Chapel Hill, Chapel Hill, NC USA; 6 Department of Genetics and Genomic Sciences, Icahn School of Medicine at Mount Sinai, New York, NY USA; 7 Department of Medicine, Icahn School of Medicine at Mount Sinai, New York, NY USA; 8 Colorado Center for Personalized Medicine, University of Colorado Anschutz Medical Campus, Aurora, CO USA; 9 Department of Biostatistics and Informatics, University of Colorado Anschutz Medical Campus, Aurora, CO USA

**Keywords:** Genome informatics, Population genetics

## Abstract

The ability to identify segments of genomes identical-by-descent (IBD) is a part of standard workflows in both statistical and population genetics. However, traditional methods for finding local IBD across all pairs of individuals scale poorly leading to a lack of adoption in very large-scale datasets. Here, we present iLASH, an algorithm based on similarity detection techniques that shows equal or improved accuracy in simulations compared to current leading methods and speeds up analysis by several orders of magnitude on genomic datasets, making IBD estimation tractable for millions of individuals. We apply iLASH to the PAGE dataset of ~52,000 multi-ethnic participants, including several founder populations with elevated IBD sharing, identifying IBD segments in ~3 minutes per chromosome compared to over 6 days for a state-of-the-art algorithm. iLASH enables efficient analysis of very large-scale datasets, as we demonstrate by computing IBD across the UK Biobank (~500,000 individuals), detecting 12.9 billion pairwise connections.

## Abstract (web summary)

Traditional methods to identify genomic regions identical-by-descent (IBD) do not scale well to biobank-level datasets. Here, the authors describe a new IBD algorithm, iLASH, which uses LocAlity-Sensitive Hashing to provide rapid IBD estimation when applied to the PAGE and UK Biobank datasets.

## Introduction

Inferring segments of the genome inherited Identical-By-Descent (IBD) is a standard method in modern genomics pipelines to understand population structure and infer relatedness across datasets^1–6^. Furthermore, it can be leveraged for alternative mapping strategies such as population-based linkage^7^, capturing rare variation from array datasets^8^, and improving long-range phasing^9^. However, the ability to scale this process to mega-scale datasets while comparing individuals along the genome has been limited. While approximate methods have been developed to improve phasing^9–11^, the identification of accurate segments inherited IBD has been limited, making its integration with variant-based testing challenging in the modern genomic context.

Here we extend ideas originally applied to large-scale similarity detection^12^ to develop iLASH, IBD by LocAlity-Sensitive Hashing, a novel algorithm that provides ultra-rapid and sensitive computation of identity-by-descent. In contrast to previous methods, which are based on conventional hashing techniques (e.g., GERMLINE^13^), iLASH uses a locality sensitive hashing (LSH) technique on slices of genotype array data. A slice is a genomic segment with the same boundaries across all the individuals. The length of the slices is generally set to be close to the minimum length of the IBD tracts being searched for (e.g., 3 cM). iLASH identifies with high probability whether the DNA of two individuals in a slice is IBD, while at the same time removing the vast majority of non-matching pairs from consideration, which accounts for its efficiency. iLASH, then, combines matching slices and extends their boundaries to determine the full extent of an IBD tract between each pair of individuals.

## Results

The iLASH algorithm is a modification of the LSH algorithm designed expressly for identity-by-descent detection on genomic data. LSH algorithms are a category of hashing functions that preserve distances while maintaining computational efficiency in high dimensional applications^14^. LSH algorithms have been shown to be efficient in machine learning^15^, entity linkage^16^, search engines^17,18^, and other disciplines^15,19^. While common hash functions map the vectors in domain space to random vectors in target space, LSH algorithms map nearby vectors in the domain space to nearby, or identical, vectors in target space^17^. This tolerance for small errors makes LSH algorithms more suitable for dimensionality reduction of genetic data without sacrificing accuracy compared to common hash functions. Moreover, since iLASH identifies the similarity of relatively large regions (slices) at a time, it provides significant speed-ups compared to simpler hashing methods. Specifically, GERMLINE uses one level of hashing with short seeds to find possible candidate matching pairs, which are later extended to larger segments. Since the seeds are relatively short to ensure sensitivity, there is significant probability that the match will not extend to the desired minimum length (e.g., 3 cM, which could be hundreds of SNPs in dense regions). Those candidate matches would increase runtime without yielding any true matches. In contrast, iLASH uses LSH that can identify, with high probability, whether two individuals match over the whole segment of interest (i.e., the whole slice size, which is usually set to values close to the desired IBD length, e.g., 3 cM). Therefore, iLASH generates significantly fewer possible candidates, each of which has a high probability of being a match, and wastes relatively little time on analyzing ultimately unsuccessful match candidates. We provide further speedups via a careful implementation that leverages multiple processing cores, now commonly available in modern CPUs, and through parallelizing computation in multiple stages of the algorithm. This parallelization also takes advantage of idle cycles during file reading and writing using multithreading programming libraries. Overall, a combination of algorithmic design and low-level optimizations allows for increased efficiency in large-scale genomic investigations.

**Fig. 1:**
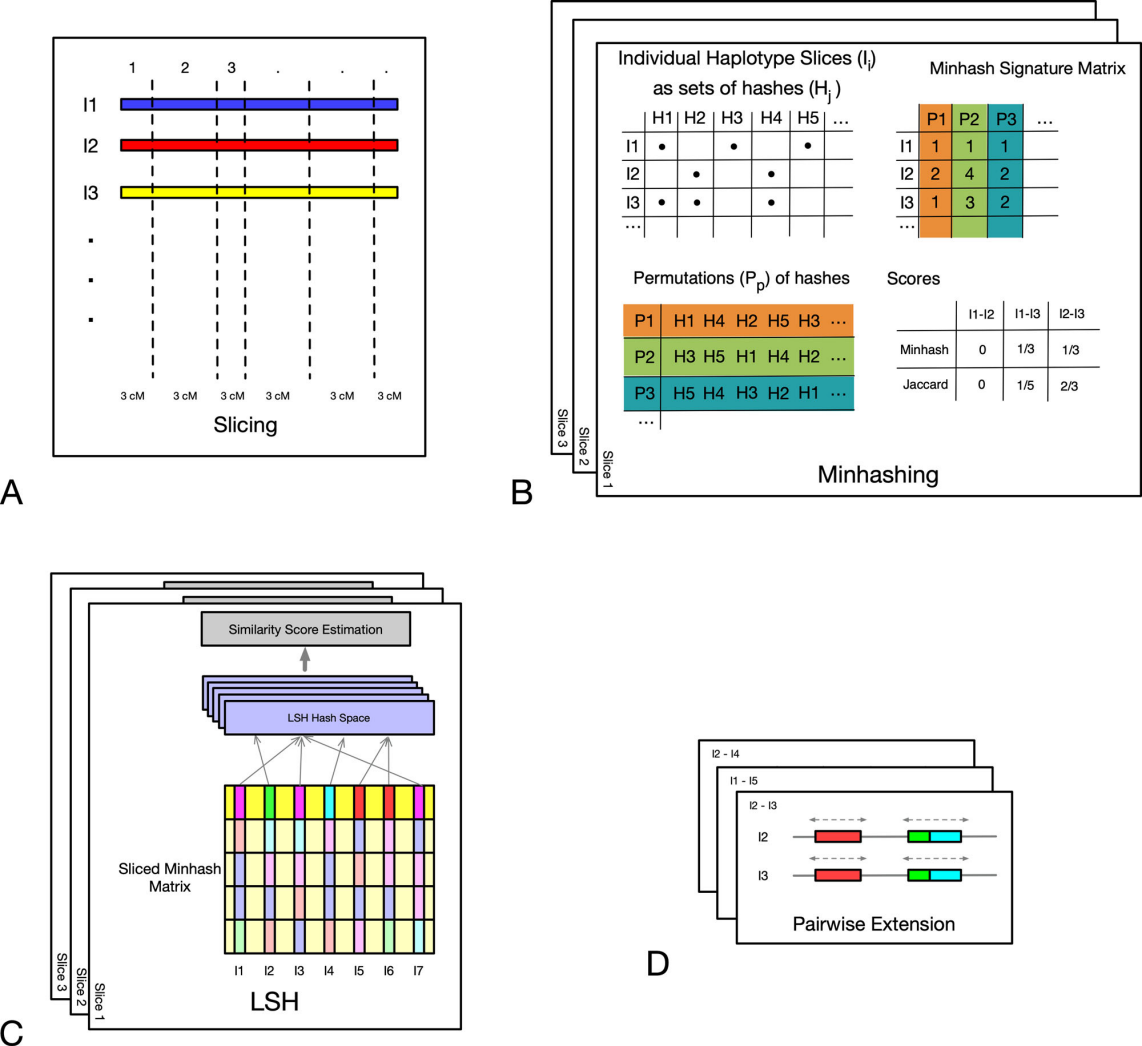
iLASH pipeline. Schematic of the iLASH algorithm pipeline. Starting from the top left with the *Slicing* step (**A**) where haplotypes are broken into slices (segments of uniform or variable length). The *Minhashing* step (**B**) creates minhash signatures by generating a table of random permutations. The *LSH* step (**C**) bands together minhash values to create an integrated LSH hash table where candidate matches are grouped together. Finally, in the *Pairwise Extension* step (**D**), these candidates are further analyzed to be extended in the (likely) case that an IBD tract spans multiple windows.

The framework of the iLASH algorithm is described next and is shown in Fig. 1 with additional details available in the Methods section. The algorithm relies on phased haplotypes from a typical GWAS array with hundreds of thousands to millions of variants (SNPs) represented as binary strings (with 0 as the major allele, and 1 as the minor allele). We are only interested in the similarity of segments longer than a given threshold, as the probability of a shared haplotype being IBD increases as segments grow longer^13,20^. Therefore, the first step of iLASH is to break the genetic data of the population into slices of a prespecified genetic length around the threshold of interest, say 3 cM (Fig. 1A). Each slice can be processed in parallel until the last step of the algorithm. To maximize efficiency, iLASH uses idle input time while reading the genotype data. Second, iLASH breaks each slice into segments of *k* bits (aka *k-mers*, or *shingles*, with *k* typically 10 to 30 bits), either in a contiguous or overlapping fashion. The genetic data for all the individuals in a slice is then transformed into sets whose elements are the distinct k-mers. iLASH uses the Jaccard similarity^21^ between these k-mer sets as a means to detect IBD. The Jaccard similarity between two sets is simply the size of their intersection divided by the size of their union. Formally, given two sets S1 and S2, ${Jaccard}\left(S1,S2\right)=\frac{{|S}1\cap S2|}{{|S}1\cup S2|}$. A large proportion of k-mers being shared between (the k-mer sets in a slice for) two individuals can be interpreted as a higher chance for them to share an IBD segment on the slice.

In the third step, iLASH computes the *minhash signature*^16^ of these k-mer sets as illustrated in Fig. 1B.^22^ The minhash approach provides a very efficient way of approximating the Jaccard similarity between all pairs of sets using a sublinear number of comparisons, efficiently scaling in large datasets. To create the minhash signature matrix, iLASH generates random permutations of the k-mers in a slice, and for each permutation it records the first index at which an individual’s shingle set at that slice contains a value, called the minhash value of the individual for that permutation at that slice. For example, in Fig. 1B for individual I3 and permutation P2 (H3, H5, H4, H1, H2), the first element in the k-mer set I3 following the order of permutation P2 that has a non-empty value is H4, which is the 3rd element of the permutation, thus the minhash(I3,P2)=3. The probability of two individuals having the same minhash values (for each permutation) is equal to the Jaccard similarity of their k-mer sets. Hence, the Jaccard similarity can be estimated by generating minhash values using different permutations and comparing them; with the estimation error decreasing as the number of permutations increases. For example, even with the low number of 3 permutations in Fig. 1B, the intersection of minhash signatures and the Jaccard similarity of I1 and I2, and I2 and I3 coincide (though it is an overestimate for I1 and I3). iLASH efficiently computes the permutations using hashing functions, which enable computing the minhash signatures in parallel using multiple threads. However, it would be computationally inefficient to compare all pairs of minhash signatures. Thus, in its fourth step, iLASH uses the LSH banding technique to generate LSH signatures (Fig. 1C). It then uses a synchronized hash table to find LSH signatures common between two individuals efficiently. iLASH allows for significant speedup by only selecting candidates for comparison that have an increased probability of having a high Jaccard similarity and, as a consequence, avoiding most of the possible comparisons. Specifically, iLASH groups the minhash signatures into b bands, comprised of r minhash values, and hashes each band. Assuming that the Jaccard similarity of I1 and I2 is s, it can be shown^22^ that the probability that two individuals agree in at least one LSH hash value is 1 - (1 - s^r^)^b^. This is a logistic function with a step transition controlled by parameters r and b, which can be tuned to trade-off accuracy of approximation versus efficiency. Finally, iLASH uses these similar LSH hashes to find candidate IBD matches and then examines the neighboring slices of a match candidate pair to extend the matched region to the full IBD segment (Fig. 1D, rightmost pane). To further increase efficiency, iLASH uses idle output time for calculating the extended IBD tracts, their length and exact similarity.

iLASH takes phased genotype files in plink^23^ format as input and its source code is publicly available^24^. For efficiency purposes, iLASH is implemented in C++. To foster usability, iLASH is designed to run on a single machine with one command with defaults optimized over multiple simulation scenarios. However, it is highly configurable to allow users to tune its behavior for each dataset (e.g., arrays of different densities). Configuration details appear in the Methods section and in the iLASH code repository.

We present a thorough evaluation of iLASH performance for both simulated data and real data from the PAGE consortium and the UK BioBank. We compare iLASH with GERMLINE^13^ and Refined IBD^25^ for both performance and accuracy. GERMLINE is similar to iLASH in that it uses hashing methods for IBD inference, although iLASH LSH hashing is more complex. Refined IBD combines GERMLINE hashing approach and Beagle Hidden Markov Models (HMM) algorithms. While HMMs can help with the task of estimating IBD, the size of our test datasets is intractable for some of the tools utilizing it, such as PLINK, so we do not compare with them at scale. We also separately compare iLASH against RaPID^26^, a recently developed scalable IBD algorithm, for both performance and accuracy.

### Performance on simulated data

To investigate iLASH performance, we simulated IBD haplotypes for three separate populations with different average IBD and for sizes ranging from 1000 to 80,000 individuals. To create these data, we first used HAPGEN2^27^ to simulate 80,000 individuals on chromosome 1 (with 116,415 SNPs) with an elevated error rate in the copying model (with the error rate for a given SNP, Θ = 130) to decrease background IBD. Then, we scanned three populations in the PAGE dataset with different cryptic relatedness characteristics: African American (low IBD on average), Puerto-Rican (a founder population with elevated IBD sharing), and all the individuals in PAGE. We extracted the detailed IBD distributions in these populations in order to generate “ground truth” populations, that is, simulated populations with the same number of segments and lengths observed in the reference populations among any randomly selected group of 1000 samples. We repeated this process to create a ground truth IBD map for 80,000 samples. The Puerto Rican population IBD simulation, for example, has more than 10 million shared segments with a total length of 62 million cM.

### Accuracy

**Fig. 2:**
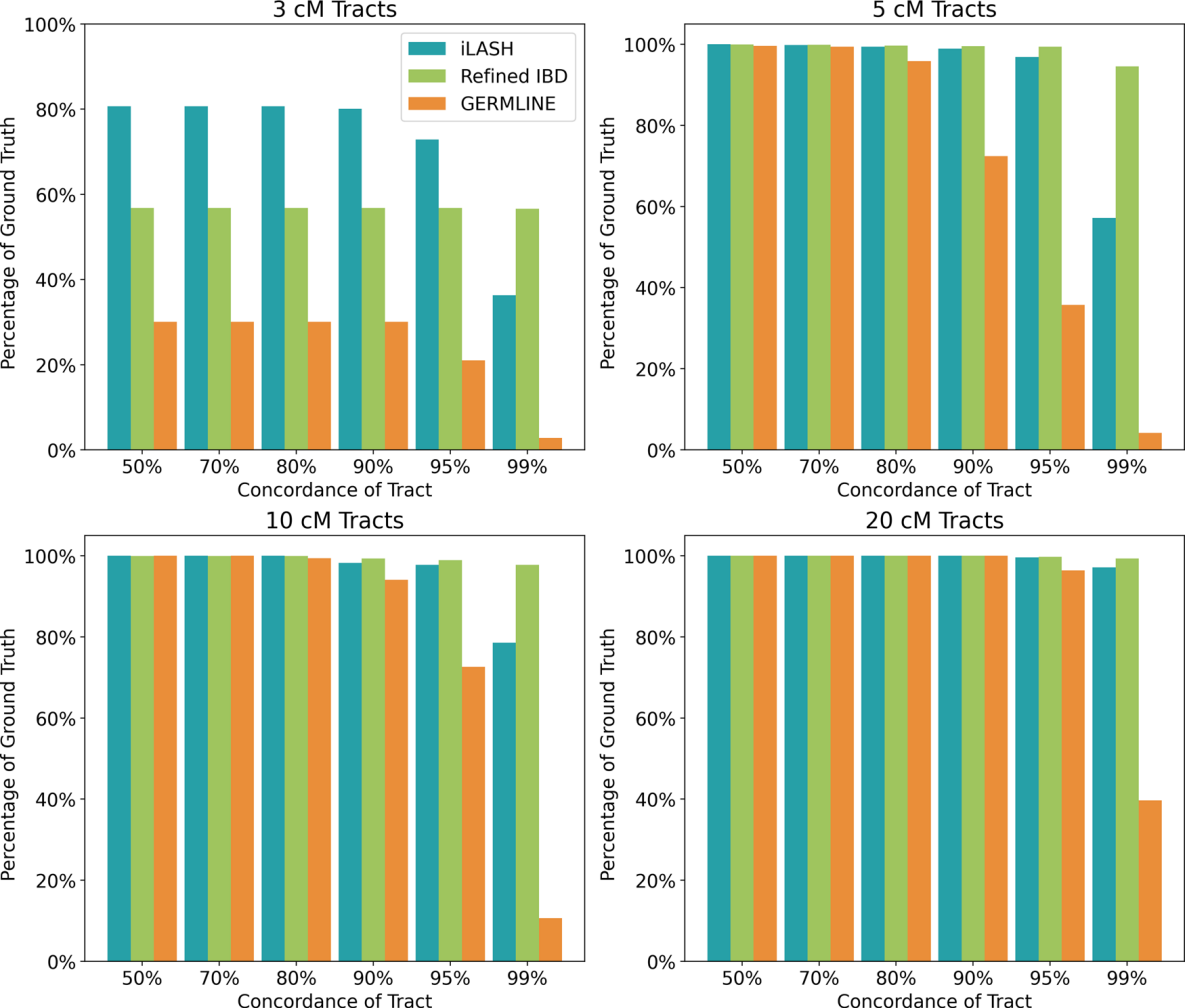
Accuracy of IBD estimation tools in simulated data. Accuracy of iLASH, GERMLINE, and Refined IBD on simulated data (30000 samples derived from the Puerto Rican population in the PAGE study sharing 3,660,900 IBD segments) at tract lengths of 3, 5, 10, and 20 cM and accuracies from 50 to 99%. The displayed percentages are based on the total number of IBD tracts with the specified length. Source data are provided as a Source Data file.

Using our simulated data as ground truth, we compared the accuracy of iLASH, GERMLINE, and Refined IBD. Here, we define the accuracy for a ground truth segment as the portion of the segment that an IBD algorithm recovers. For example, if for a ground truth segment of 5 cM, iLASH identifies 4 cM, then we say that the accuracy of iLASH is 80% for that segment. We report the cumulative accuracy across all IBD segments tested (i.e., the total length of all segments identified by each algorithm divided by the total length of ground truth IBD segments), and the accuracy for segments at different lengths (3 cM, 5 cM, 10 cM, and 20 cM). Overall, iLASH accurately recovers at least 96% of the total length of all simulated IBD haplotypes across the simulated dataset of the three populations tested, compared to lower overall accuracies of 94% for Refined IBD and 85% for GERMLINE. These results were consistent for dataset sizes ranging from 1000 to 30,000 individuals for all three algorithms (cf. Supplementary Fig. 1). However, among individual segments, the accuracy varies significantly with the tract length, as shown in Fig. 2, with iLASH performing similar to or better than Refined IBD, except for the highest level of accuracy (99%) on tracts shorter than 10 cM. Both iLASH and Refined IBD outperform GERMLINE both on shorter tracts and on longer tracts when high accuracy is required. For example, for tracts of 3 cM, iLASH identifies ~80% of tracts with at least 90% accuracy (i.e., 90% of the ground truth segment recovered), while Refined IBD and GERMLINE identify 57% and 35% of those tracts respectively. iLASH identifies close to 100% of the of 5, 10, or 20 cM long tracts with at least 95% accuracy.

Overall, all three tools recover the majority of ground-truth IBD existing in each individual (cf. Supplementary Fig. 11). Stratifying by bins of tract length and IBD accuracy demonstrates more nuanced features of performance. While iLASH and Refined IBD show similar accuracy for segments > = 5cM, iLASH shows higher accuracy for detecting smaller segments (3 cM). Refined IBD performs more accurately when finding the exact borders of segments, when an accuracy of higher than 99% is required. However, Refined IBD has a lower recall, missing portions of IBD ground truth segments when these are short. For example, for 3 cM segments at 90% accuracy, iLASH identifies 80% of the ground truth segments, while Refined IBD only identifies 57% of these segments. Compared with the other two methods, GERMLINE underestimates the IBD at higher accuracy and shorter tracts.

### False positive rate

To investigate the rate of false positives of iLASH, Refined IBD, and GERMLINE, we took a composite-individuals approach^28^. We used chromosome 2 data (115 K SNPs) from 2000 sampled individuals from the African American population of the PAGE study. In order to minimize the possibility of false positive IBD inference, we divided the genotype data into slices of 0.2 cM length, and we shuffled these slices among the 2,000 individuals. Before this process, iLASH was able to find 10,459 IBD tracts in the dataset. After shuffling, iLASH finds only 1 tract (of 3.9 cM), while Refined IBD finds 98 (false positive) tracts, and GERMLINE finds 27,838 (false positive) tracts, with 99.9% of them around a low complexity region starting from base pair 2766642 and ending at base pair 3042093, which contains only 60 SNPs on the PAGE array data (cf. Supplementary Fig. 2). We repeated this experiment 5 times. The average number of false-positives segments for iLASH, Refined IBD, and GERMLINE was 2.2 (standard deviation = 2.23), 93.4 (standard deviation = 7.2), and 25,779.4 (standard deviation = 641.2), respectively. Thus, the false-positive rate of iLASH in this experiment is ~2% and 0.008% of those of Refined IBD and GERMLINE on average. The average length of the 11 false-positive segments found by iLASH across all 5 experiments was 3.51 cM (standard deviation = 0.259); while the average length of false-positive tracts found by Refined IBD and GERMLINE were 3.7 cM (standard deviation = 0.44) and 3.96 cM (standard deviation = 0.037) respectively. All of the false-positive segments found by iLASH, Refined IBD and GERMLINE were on the same region of low complexity (cf. Supplementary Fig. 2).

To evaluate the effect of SNP array density, we dropped one out of every 3 SNPs in the same dataset as above. In the less dense array, the number of tracts found by iLASH decreased to zero. Refined IBD and GERMLINE results also decreased to 85 and 1097 segments, respectively, all of which were still in the same low-complexity region as before. When we increased the size of reshuffled slices to 0.5 cM, iLASH identified 3 tracts for the normal data, and 13 tracts for the data with lower SNP density. Refined IBD found 960 and 880 tracts, and GERMLINE identified 22,688 and 26,724 tracts for the dense and trimmed haploid files, respectively. More than 99% of the false positive tracts found by GERMLINE and Refined IBD were located in the same low complexity region described above. In contrast, iLASH showed near perfect performance across different regions and array densities.

To evaluate the performance in regions of high complexity, we tested the tools on chromosome 6, using the same data generation method and on the same dataset. None of them found any false positives over ten repeated experiments. Since all three tools use phased haplotype data instead of genotype data, their precision is dependent on the accuracy of the phasing stage, however standard phasing algorithms embrace approximate IBD inference to improve long-range phasing. Such methods are expected to improve phasing accuracy in large studies, particularly in the haplotypes spanning IBD segments. We used a threshold of 3 cM to conduct these experiments (on chromosomes 2 and 6). This threshold was selected based on the density of the array which limits the amount of information available at lower lengths.

### Runtime and memory

To compare the time efficiency of iLASH against Refined IBD and GERMLINE, we used the same simulated datasets as the previous section. We ran the tools on the same machine, a workstation running CentOS Linux release 7.4.1708 with 128 GB of memory and Intel® Xeon® Processors E5-2695 v2 with 12 cores and 24 threads on it using 30 MB of shared cache. Both iLASH and GERMLINE are implemented in C++, but in order to use a specific version of the Boost library, GERMLINE required an earlier version of the C++ compiler. Refined IBD is implemented in Java.

**Fig. 3:**
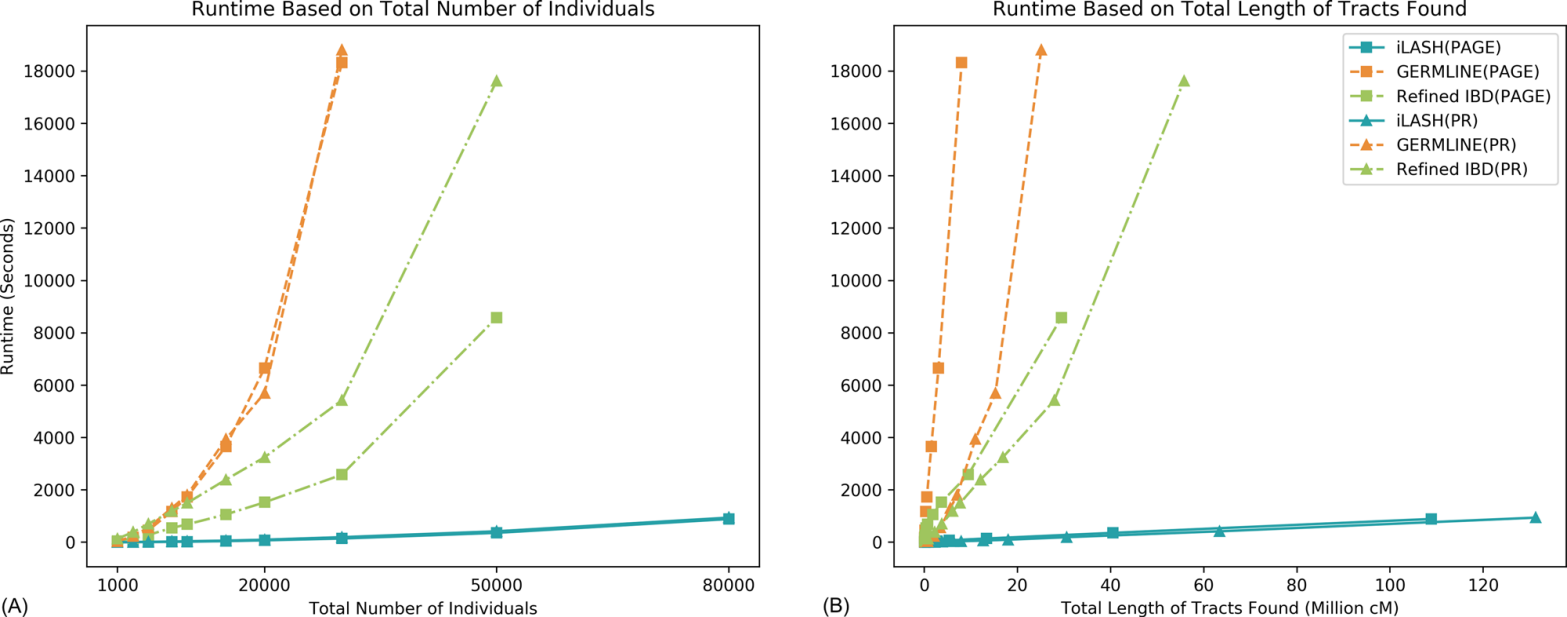
Comparison of runtimes among algorithms. IBD computation runtime in seconds for iLASH, Refined IBD, and GERMLINE on synthesized haplotypic data simulating all of PAGE and Puerto Rican (PR) populations IBD patterns: (**A**) as the number of individuals grows, (**B**) as the total output (total length of tracts found) grows. Source data are provided as a Source Data file.

Figure 3A shows the computation time in seconds for iLASH, Refined IBD, and GERMLINE as the population size grows. The largest dataset on which we were able to run GERMLINE tractably on one machine contained 30,000 individuals. It took GERMLINE over 5 h to analyze this dataset. For the same data, iLASH took 3 min and 15 s. Refined IBD scaled up to 50,000 samples for which it took 4 h and 54 min. iLASH took 7 min for the same dataset. Our maximum-sized simulation of 80,000 individuals could be scanned and analyzed by iLASH in less than 16 min. Figure 3B shows the computation time in seconds for iLASH, Refined IBD, and GERMLINE as the total size of found IBD grows. iLASH exhibits quasi-linear runtime growth with the number of individuals, as well as with the total amount of IBD found. However, GERMLINE and Refined IBD runtimes deviate from linearity in populations with higher levels of IBD such as the PAGE Puerto Rican population.

iLASH has a smaller memory footprint than both Refined IBD and GERMLINE as the sample size grows (cf. Supplementary Fig. 3). Experimentally, the memory usage for iLASH and GERMLINE increases in a quasi-linear fashion with the number of individuals, However, iLASH memory usage grows at a significantly lower pace. Specifically, from 1000 to 20,000 individuals GERMLINE memory footprint grows from 880MB to 29.8 GB, while iLASH grows from 235 MB to 5.8 GB. The memory usage of Refined IBD does not follow an obvious pattern for the analyzed range. While it starts higher than the other two systems at 4.5 GB for 1000 individuals, its memory usage ends up at 15.92 GB for 50,000 individuals, very similar to the 15.86 GB memory usage of iLASH.

Array density and the desired minimum IBD length also affect runtime and memory usage of iLASH. Based on our experiments, an increase in the density of SNPs contributes to a quasi-linear increase in memory usage and runtime (cf. Supplementary Fig. 4). Specifically, on 5000 samples of the simulated data derived from Puerto Rican population of the PAGE study, from a 50% downsampled array to the full array for Chromosome 1 (116,415 SNPs) the runtime ranges from 12.7 to 14.6 s, and the memory usage ranges from 1.7 GB to 2.38 GB (average over 10 experiments).

We also found larger thresholds for minimum size of IBD segments to result in lower runtime and memory usage (cf. Supplementary Fig. 5). Specifically, for 40,000 individuals from the PAGE simulated data (Chromosome 1, 116,415 SNPs), from a 3 to a 20 cM minimum IBD thresholds, the runtime ranges from 223 seconds to 34 s, and the memory from 22.5 GB to 2.8 GB (average over 10 experiments).

### Comparison with RaPID

Recently, Naseri et al.^26^ developed an IBD detection method that relies on the Positional Burrows-Wheeler Transfom^29^ to efficiently scale haplotype matching. We evaluated RaPID on the same simulated data used in our comparisons with GERMLINE and iLASH. While RaPID is substantially faster than GERMLINE and Refined IBD, it remains slower than iLASH on all tested datasets, and the difference is particularly noticeable as the sample size increases (cf. Supplementary Fig. 6), possibly because RaPID is limited to single-thread analyses. For 1000 samples, iLASH takes 4 s, while RaPID takes 27 s. For 80,000 samples iLASH takes 15 min, while RaPID takes 98 min. More importantly, iLASH remains more accurate in our simulations: iLASH recovered over 95% of the total length of ground truth IBD across all simulations, where RaPID only recovered 72%. Short IBD segments (5–3 cM) were particularly challenging for RaPID, which generated a larger number of false positives: 22–25% of the results across runs contained discordant genotypes between identified pairs; an issue that never occurs with GERMLINE and iLASH. In spite of RaPID being a haploid method, the program does not report haplotype phase information in its output, which can constrain the options possible in downstream analysis after IBD estimation. Given these limitations and our focus on haploid-oriented metrics, our primary comparisons remain with other IBD estimation methods.

### Performance on real data from the PAGE Study

We investigated iLASH and GERMLINE IBD inference over two existing datasets: a multi-ethnic population of *N* = 51,520 individuals from the Population Architecture using Genomics and Epidemiology (PAGE) consortium, and the *N* ~ 500,000 individuals in the UK Biobank dataset. In PAGE, iLASH uncovered a total 202,424,985 segments, while GERMLINE identified a total of 195,577,460 tracts. The overall concordance between iLASH and GERMLINE was 95%. iLASH total runtime was 58 min on a single workstation (same as above) requiring between 3 GB (for chromosome 22) and 17 GB of memory (for chromosome 2). GERMLINE could not be run on the same workstation, because it required more than 128 GB of memory for every chromosome. GERMLINE was run on a High-Performance Computing Cluster at the Icahn School of Medicine at Mount Sinai, which has several high-memory nodes. For the largest chromosome (12) that could be analyzed by GERMLINE without splitting the population, GERMLINE took 6 days of computation. For the same chromosome in the single machine described above, iLASH took 3 min and 12 s, an improvement of four orders of magnitude.

**Fig. 4:**
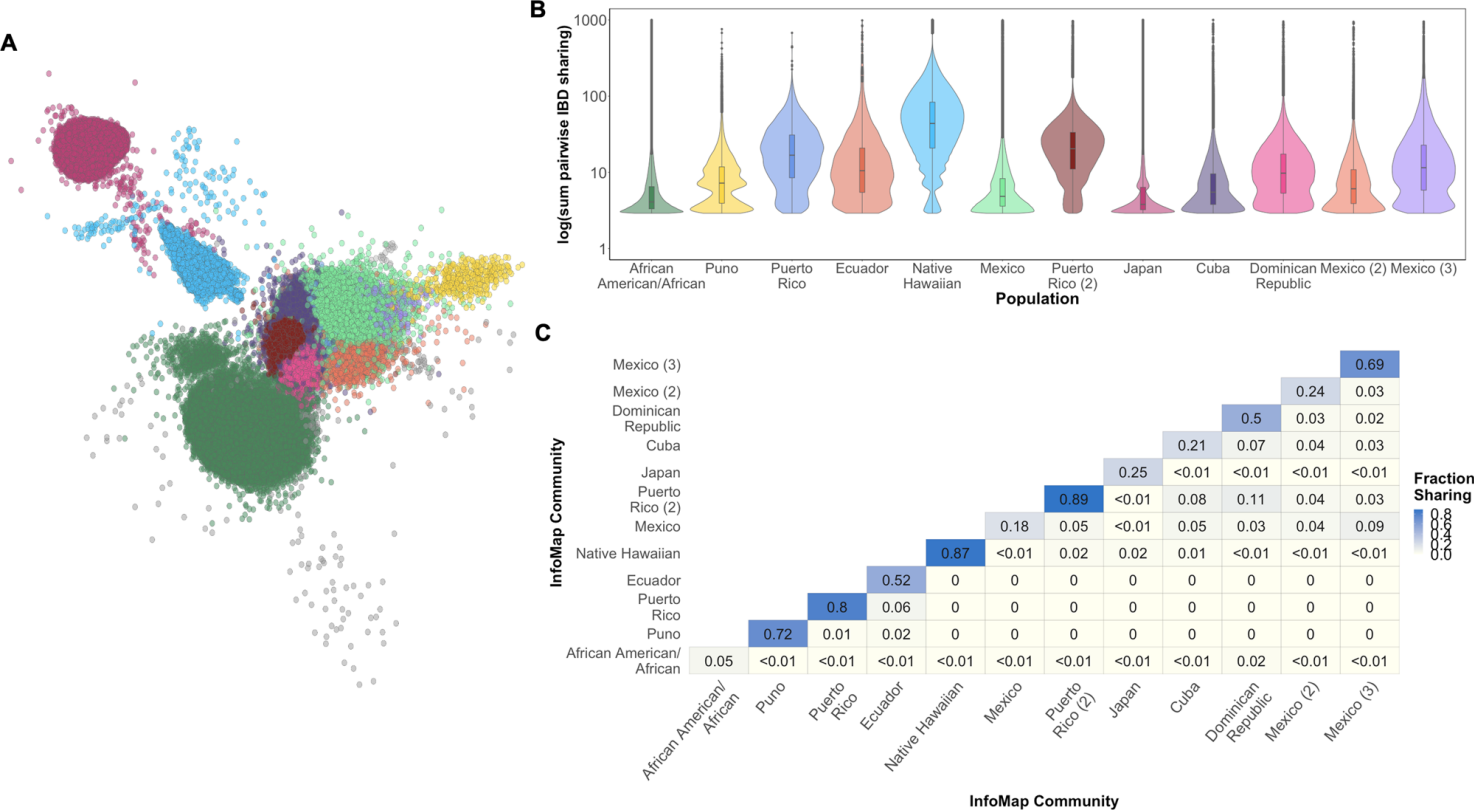
Network of IBD sharing in the PAGE dataset. **A** A network of IBD sharing within PAGE plotted via the Fruchterman Reingold algorithm. Each node represents an individual (edges not shown). Individuals are colored based on community membership as inferred by the InfoMap algorithm. **B** Distribution of the sum of IBD sharing within the top 16 largest InfoMap communities demonstrates variance in levels of IBD sharing between different communities. Boxplots inlayed within violins depict the median and interquartile range of the within-community sum of pairwise IBD sharing (cM), while the minimal and maximal values per distribution are represented by the extreme tails of the violin plot. InfoMap communities are labeled according to the demographic label that most strongly correlated with community membership (as measured by positive predictive value). Elevated pairwise IBD sharing can be observed in several InfoMap communities, which may represent founder effects. **C** Heatmap of the population level fraction of IBD sharing within and between the top 16 largest InfoMap communities demonstrates elevated sharing within, relative to between communities.

To explore the utility of IBD haplotypes inferred by iLASH in a large genomic dataset we constructed an IBD-based network of distant relatedness among the PAGE dataset^30^. In this network, individuals are represented by nodes (*N* = 38,919 across 3 PAGE Studies: WHI, MEC, and HCHS/SOL) that are connected by edges (*E* = 55,069,482) if they share any haplotypes IBD. We used this graph to explore fine-scale population substructure by applying the community detection algorithm InfoMap^31^ to the IBD network in order to uncover communities of individuals who were enriched for recent, shared ancestry in the form of elevated IBD sharing^32^. We observed that 92.3% of selected PAGE participants fell into one of 12 inferred IBD communities, each containing *N* > 100 individuals, with the remaining 7.7% of participants being assigned to communities ranging from *N* = 1 to *N* = 91 participants in size (cf. Fig. 4A). We observed that IBD community membership correlated strongly with available demographic information in the form of both self-reported ethnicity as well as sub-continental and country level region of origin (cf. Supplementary Table 1). For example, membership of one InfoMap community was highly correlated with being born in Puerto Rico (PPV 0.96), while another was correlated with being born in the Dominican Republic (PPV 0.98). We also observed significant differences in the distribution of total pairwise IBD sharing between communities (Fig. 4B). Examination of the population level fraction of IBD sharing within- and between- communities revealed a high degree of network modularity, or elevated sharing within communities relative to between (Fig. 4C). Three distinct communities emerged that correlated with being born in Mexico (PPVs 0.96, 0.43, and 0.99, respectively), the latter of which exhibited elevated IBD sharing relative to the former two and may represent a founder population of (indigenous American) Mexican origin. This analysis demonstrates the utility of IBD inference for exploring fine-scale population substructure within large datasets. Further, this elevated IBD signature empowers techniques in founder populations such as IBD mapping and detection of highly drifted alleles.

### Detecting identity-by-descent in the UK Biobank

**Fig. 5:**
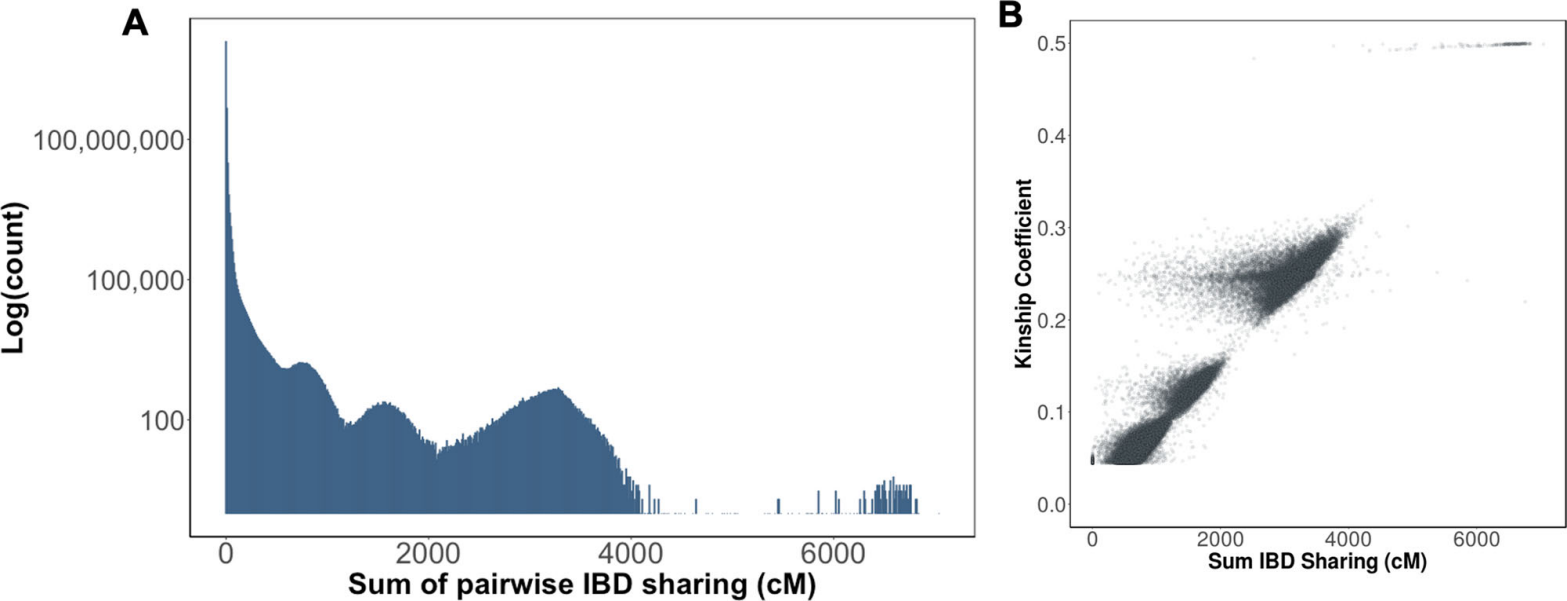
Identity-by-descent sharing in the UK biobank. **A** Distribution of the sum of pairwise IBD sharing (cM) in the UK Biobank across all *N* = 487,330 participants. **B** Correlation between the sum of IBD sharing and kinship as measured by the KING software in all pairs of individuals reported in the UK Biobank output to be > = 3rd degree relatives.

To explore fine-scale population substructure in the UK Biobank we leveraged the phased genotype data at 655,532 SNPs for *N* = 487,330 participants. We used iLASH to infer pairwise IBD segments (> = 2.9 cM) between all individuals. We observed 10.84% of all possible pairs of individuals shared at least one haplotype of their genome IBD, representing 12,867,760,228 pairwise connections in total (Fig. 5A). To understand how well the IBD sharing estimates correlated with genetic relatedness, we calculated the correlation between the kinship coefficient and the sum of IBD haplotype sharing among 3rd degree and above relationships in the UK Biobank. We observed a high degree of correlation between the two estimates (*R*^2^ = 0.95; Fig. 5B). Beyond this close relatedness, we observed 778,822 pairs of individuals exhibiting relatively high levels of relatedness (>100 cM), and additionally 43,205,248 pairs of individuals with sharing above 20 cM. In most instances these individuals were just below the level of detection as provided by the standard genetic relationship matrix released alongside the UK Biobank genotype data. However, we also identified 4,808 highly-related pairs of individuals (> = 500 cM) that were not reported to be 3rd degree relatives or above in the default UK Biobank release. To investigate this further, we replicated the KING relatedness estimation for this subset of participants, and noted that the majority of these pairs did exhibit elevated relatedness (mean kinship = 0.037, Interquartile Range = 0.031–0.043), but that their KING estimates fell slightly below the cut-off for 3rd degree relatives (>0.0442). However, some discordance between the two metrics persisted. Specifically, we identified a subset of pairs (*N* = 203 pairs, comprised of *N* = 378 unique individuals) with high IBD (>500 cM), but low or negative kinship (<0.02). We noted that levels of autozygosity within the individuals comprising these pairs was significantly elevated relative to the population average in the UK Biobank, with the mean sum of runs of homozygosity (ROH) within discordant pairs being 116.5 cM (95% C.I = 98.2–135.0 cM), compared to 1.84 cM (95% C.I = 1.79–1.89 cM, Wilcoxon *p* < 6.3e–204) in the UK Biobank overall. We speculate that this elevation of autozygosity may have contributed to the deflation of the KING kinship estimates and resultant discordance with iLASH.

Overall, we highlight in the UK Biobank that detectable relatedness exists across far more pairs of individuals than is present in the kinship matrix currently provided with the standard data release. The sensitivity of methods like iLASH to detect true levels of cryptic relatedness is critical in mixed effects models such as SAIGE^33^ and BOLT-LMM^34^ for efficient, appropriately-calibrated association testing.

## Discussion

Here we present iLASH, an accurate and computationally efficient method for detecting identity-by-descent segments. iLASH scales to large, biobank-level datasets, empowering downstream analysis that uses IBD for population genetic inference and disease mapping. In addition, we demonstrate that, consistent with population genetic theory^35^, IBD is a ubiquitous component of large-scale population datasets and can provide estimates of relatedness useful in both medical and population genetic contexts. We have run iLASH on large datasets such as the PAGE Study (*N* = 51,520) and the UK Biobank (*N* = 487,330), providing additional context to the understanding of population structure using typical measures of global ancestry relying on unlinked genotypes. As IBD breaks down via recombination across a relatively small number of generations, the observed patterns from iLASH provide a snapshot of relatedness and population structure driven by more recent patterns, rather than the more ancient patterns determined by genetic drift.

In contrast to previous methods, we gain significant performance improvements by basing iLASH on locality-sensitive hashing (LSH), an algorithm that leverages the speed of hash-based methods while allowing for some incomplete matches, whether due to genotyping or phase-switch inconsistencies. Critically, iLASH applies LSH to relatively large slices of DNA identifying with high probability whether two individuals in that slice are in IBD, and more importantly whether those individuals are unlikely to be IBD at the desired minimum threshold. This results in a small set of candidate pairs to be checked for extension and is largely responsible for the significant speed-ups of iLASH. By contrast, GERMLINE, the previous industry standard for large-scale inference, identifies IBD candidates using small hash seeds, followed by SNP-wise extension. However, many of the short hash matches may not yield a match at the desired minimum IBD length. Thus, GERMLINE incurs a greater runtime penalty, since the pairwise comparisons grow quadratically. Keeping most of our algorithm within the standard LSH framework allows our runtime to grow much more slowly in the number of individuals. In addition, we used a minimum length threshold of 3 cM for IBD estimation across our analyses in this manuscript because using hash-based algorithms such as iLASH, GERMLINE, and RaPID, to estimate short IBD segments (<1 cM) can result in an excess of false-positive edges with short segments, e.g.,^36^. However, Saada et al. have recently proposed FastSMC^37^, an IBD detection algorithm that uses coalescent based likelihood estimates to assess the validity of shorter segments. Development of a scalable pipeline where candidate matches found by iLASH’s LSH algorithm are evaluated and scored via the FastSMC method would yield a fast and more accurate approach for short segments. As demonstrated by Saada et al. using their own hash-based algorithm, GERMLINE2, these IBD tracts can then be used for downstream analyses, such as inference of fine-grained population structure, evolutionary processes, and rare variant association tests.

While this windowed hash-based method could mean that our method is less precise at identifying IBD endpoints along the chromosome, in practice, our simulations show otherwise. We validated this method via simulations, ensuring that we could both recover short segments (3–5 cM) as well as the full length of longer segments, allowing for downstream utility of the IBD tract length distribution. Our method is far more sensitive than GERMLINE at identifying short segments, an observation found by others^38^. Identifying large segments is critical for inferring an unbiased tract length distribution, an observation required for IBD-based genetic relatedness matrices^39^, as well as population genetic inference^3^. While maintaining high sensitivity in short segments, we ensure that our method is not susceptible to false positives via genome shuffling^28^ to create a putatively IBD-free dataset. Consistent with our method being haplotypic, false positives should not be an issue, and we observe our method being almost entirely without false positives, up to the detection limit of haplotypic complexity in our test data. We note that these false positive tests can be highly dependent on the diversity present in the population used for simulation, therefore we chose a population with limited endogamy, derived from PAGE Study African Americans, to test iLASH.

In addition to identifying cryptic relatedness in a dataset, we anticipate our scalable IBD tool to provide additional insights into large-scale datasets such as PAGE, UK Biobank, and larger datasets such as the Million Veteran Program and the All of Us Research Program. iLASH works within standard pipelines as it uses a standard input file format, and output from these estimates can easily be integrated into medical and population genetic workflows. As an example, we demonstrate the utility in estimating IBD segment patterns across real-world datasets, allowing for downstream population clustering using graphical methods able to tease apart fine-scale population structure at a level beyond standard SNP-based methods such as PCA. This, then, can have large implications for medical genetics, particularly at the rare end of the frequency spectrum, where variants are far more likely to be private to one or a small number of populations. For example, we have shown previously that IBD tracts allow us to identify a population-specific variant underlying a recessive musculoskeletal disease in Puerto Ricans, that could not be detected using standard SNP-based genome-wide association approaches^8^.

While our method is computationally efficient, mega-scale datasets in the hundreds of thousands to millions still benefit from data subsetting in the current version of iLASH, as we did when running iLASH over the UK Biobank. This can be ameliorated with runs on high-memory nodes. However, to fit the entirety of a dataset on a single machine will require additional data compression, likely through methods such as the Positional Burrows-Wheeler Transformation (PBWT), employed by the Sanger Imputation Service. These approaches can be integrated efficiently in the future, along with other methods, such as incremental computation of IBD as new subjects are added to large datasets, such as the evolution of the UK Biobank 150,000 participants release to the current >500,000 individuals, or the client growth in direct-to-consumer companies. A distributed implementation of iLASH, designed natively to be run over nodes in a cluster, fits well with the underlying algorithm and would allow for an even more scalable solution.

We have currently focused our methods on common variants as are typically found in genotype arrays. We plan in the future to update iLASH to account for recent, rare mutations as are present in sequence data. As our algorithm is based on locality-sensitive hashing we can easily and efficiently handle mismatches due to genotype error or recent mutation on an IBD background. This simply will require modification of haplotype similarity thresholds and SNP density. With large, diverse sequencing datasets soon available, we anticipate this as a future improvement to a new version of iLASH.

Numerous methods have been created to model population structure for large, diverse populations. However, as datasets grow, the effects of population structure become inevitable, and the relevance of demographic history influencing patterns of cryptic relatedness become unavoidable. This has particular implications for how we think of genotypic similarity. Where phasing and imputation are standard workflows, we provide a method to integrate IBD analysis into existing pipelines, allowing for novel population identification and inference of demographic history. From these we can both provide a method forward for population-based linkage as a complement to standard GWAS approaches, as well as an efficient way of identifying sub-populations within a larger dataset. Methods such as iLASH then, while having their roots firmly in early medical genetic studies, can then provide insight for the future of large-scale and multi-ethnic cohorts available in biobanks and national initiatives.

## Methods

In this section, we describe in detail the algorithm and implementation techniques used in iLASH, including parameter configurations and their effect on the performance of iLASH.

### Background and rationale

iLASH was inspired by a minhash-based realization of the LSH algorithm^16,17,22^. Locality Sensitive Hashing (LSH) refers to a category of hashing methods that preserve a specific distance function. A hash function is called “locality-sensitive” if it maps close vectors in the source domain to close or identical vectors in the target domain. A good example of such hash functions is mapping the points on the surface of a half-sphere to a 2D circle on a plane beneath them. This function reduces dimensionality from 3 to 2. However, the output of such mapping still has enough information to infer the distance among different points on the 3D curve.

LSH was developed and is often used for duplicate string detection in large text corpora^40–42^. In general, it is not feasible to compare every pair of strings, since the computational cost grows quadratically with the number of strings. Moreover, it is desirable to also identify segments that are similar, but not identical, since we need to account for text modifications such as typos, local rephrasing, insertions of advertisements, personalized greetings, or other dynamically generated text in a web page. Jaccard similarity, or the intersection of two sets divided by their union, is a measure fit for such tasks.

The LSH implementation used in finding text duplicates thus tries to preserve the Jaccard similarity between different pairs of strings.^22^ The first step is to convert each string into a set of shingles (aka *n-grams*, substrings of *n* characters; or *k-mers* in the genetic context) and conceptually create a matrix with the strings (sets) as rows and all the distinct shingles (elements) as columns. Then, *LSH* estimates the Jaccard similarity between the sets (strings) by doing two levels of hashing. The first hashing step of LSH is called *minhashing*. To create the minhash matrix, the algorithm generates *n* random permutations of shingles. For each permutation P, it records for each set S, the index of the first shingle included in S (cf. Fig. 1). The probability of two sets having the same minhash value for each of the permutations is equal to their Jaccard similarity score. The second level of hashing is called the LSH and operates on the minhash vectors. To calculate LSH signatures, consecutive minhash values are grouped together and hashed for a second time. Suppose there are n minhash values for each string, grouped in *b* bands. Each band is comprised of $r=n/b$ minhash values. Suppose S_1_ and S_2_ have a Jaccard score of *s* between them. Then the probability of all minhash values in a band being equal between the two sets is s^r^. The probability that at least one of the minhash values in a band being different is 1-s^r^. If one or more than one of the values in a band differs between S_1_ and S_2_, then the LSH signature of that band is going to be different for the two sets. Thus, the probability of all LSH signatures being distinct for each set is ${(1-{s}^{r})}^{b}$. Using this equation, we can calculate the probability of two sets sharing at least one LSH signature, leading to them being declared a hit as $1-{\left(1-{s}^{r}\right)}^{b}$. This probability distribution is a sigmoid function a step transition that can be controlled by changing values of r and b to trade off the number of comparisons and the false positive and false negative rates.

Parallels between finding similar strings and similar haplotypes make adopting LSH in the genomic domain attractive. However, applying LSH over entire chromosomes is not useful for IBD, since the goal of IBD estimation is to find exact loci shared between pairs of individuals and not an average estimation of overall similarity of pairs of chromosomes. Dividing the genotype data in segments (*slices*) of a size close to the desired minimum IBD takes full advantage of the strengths of the LSH. The similarity score of individuals sharing an IBD tract in or around those slices would dramatically differ from that of a non-sharing pair. For example, in the problem of IBD estimation for tracts of at least 3 cM in a dataset, if all the haplotypes are divided similarly into slices shorter than or equal to 3 cM each, three scenarios could happen to IBD segments of 3 cM in a database.

1. The IBD segment is located exactly within one slice’s boundaries with minimal overflow/underflow. Then, the LSH algorithm would signal almost an 100% similarity in that slice.
2. The IBD segment is spread more on one slice and less on the neighboring slice. One of the slices will have more than 50% similarity in between the two individuals and the other slice will have less than 50% shared.
3. The IBD segment is located almost half and half between two neighboring slices. Since the segment is larger than 3 cM (the length of each slice), each slice would have around 50% similarity or more.

In each of these scenarios, there will be at least one slice that has a Jaccard similarity score equal to or greater than 50% between the two individual haplotypes sharing IBD. iLASH estimates such similarities in order to find IBD tracts. Segments longer than 3 cM yield a greater overlap within at least one slice and thus have a higher minimum score between two haplotypes. By inspecting neighboring slices to a slice with a high degree of estimated similarity, iLASH finds the true extent of an IBD segment. In the following section, we discuss how using dynamic and overlapping slicing ensures that the full IBD segment is identified with high probability.

GERMLINE and RaPID also use hashing techniques to identify IBD. GERMLINE uses relatively short hashes to find candidate IBD matches. However, this results in too many candidate pairs, many of which will fail to extend to the desired minimum IBD. Since candidate pairs scale quadratically, this prevents GERMLINE from scaling up to large datasets. RaPID uses the Burrows-Wheeler transform, which effectively operates on sub-sampled genetic data. Although scalable, this method results in lower accuracy compared to iLASH or GERMLINE (cf. Supplementary Fig. 11). In contrast, iLASH conducts its (LSH) hashing on segments of a length similar to the desired minimum IBD, which has the double benefit of proposing candidate pairs that have a high probability of being in IBD (for a slice), and more importantly eliminating most pairs from consideration (which would have an extremely low probability of being on IBD). Our experiments on accuracy and false positive rate support the use of LSH in iLASH as a good tradeoff between accuracy and scalability.

### iLASH algorithm and settings

As discussed earlier, the iLASH algorithm has four main steps (cf. Fig. 1). In the first step, iLASH divides the haplotype data into segments with the same fixed boundaries (slices) across all the individuals, in order to apply LSH to each slice. One could consider *static* slices, say 2000 SNPs. However, genotype arrays are not usually sampled uniformly throughout the human genome. Thus, there can be slices of 2000 SNPs that cover a 5 cM long haplotype and there can be others covering 2 cM in the same experiment. Therefore, we use *dynamic slicing* where each slice corresponds to a fixed genetic distance of ***k*** cM, close to the desired minimum IBD length (usually 3 cM). Each slice, then, comprises a variable number of SNPs. For added precision in iLASH, we can generate slices that overlap, so that for IBD segments close the desired minimum length, there is always one slice that will show high similarity with the true IBD segment. For example, since in our experiments the desired minimum IBD was of 3 cM, we defined a slice overlap of 1.4 cM. Overlapping slices significantly increase accuracy, at a moderate computational cost. Another challenge is areas of low complexity (and thus of low array density), where a small number of SNPs could represent a long haplotype. These regions often generate an increased rate of false positives. To address this, we defined a threshold in iLASH to prevent the analysis of slices with lower than a given SNP count. For example, in our experiments we ignored slices with fewer than 50 SNPs. While we have found these parameters to yield good results on our datasets, they may or may not be suitable for other datasets. Our implementation of iLASH allows the user to configure all these parameters.

In the second step, iLASH tokenizes the SNP data in each slice into shingles (k-mers). The main effect of shingling is to turn the genetic segments into sets of hashes. In our experiments, each shingle encompasses 20 SNPs. Smaller shingle length does not necessarily mean higher precision as it may cause the number of possible values for each shingle to decrease which results in lower precision. The shingles are then mapped to a 32-bit integer space using FNV hashing (https://tools.ietf.org/html/draft-eastlake-fnv-03) to allow for uniformly representing shingles of different lengths (cf. Other Technical Notes below). No stored hash structure is used so as to maximize the speed gains by eliminating the need for a synchronized memory access procedure. By default, iLASH uses non-overlapping shingles. Our experiments used this default setting. However, the tool has the option to generate overlapping shingles which can help with noisy data and in sparse regions by increasing the similarity at the cost of a modest increase in run time.

In the third step, iLASH calculates minhash signatures for each slice. The probability of overlap between the minhash signatures of two slices matches the Jaccard similarity of the set representation of the slices. The Jaccard similarity of two slices (sets) S_1_ and S_2_ is the number of shingles they have in common divided by union of the shingles present in the slices. The minhash signatures can be seen as a compressed representation of the slices, reduced from the number of distinct shingles (hundreds to thousands) to just a few minhashes (20 in our experiments).

Finally, in the fourth step, following the LSH algorithm iLASH applies the banding technique to the minhash signatures, that is, it groups the *n* minhash values into *b* bands of *r* values each (n = b*r). Now, iLASH applies another level of (simple) hashing to these groups to find similar slices and propose candidate pairs. In our experiments on simulated PAGE data, we used 20 minhash permutations (values) per slice (*perm_count = 20*). We grouped these minhash values into 5 bands to generate LSH signatures (*bucket_count = 5*).

The choice of the number of bands (aka buckets) and permutations depend on the problem. Here we provide some guidance on how to set these values and experiments on their effect in our evaluation datasets. As we show in Supplementary Figs. 7 and 8, there are tradeoffs between accuracy (percentage of true IBD recovered), false positives, running time, and memory consumption depending of the configuration of bands and permutations per band. In our experiment with 10,000 individuals (derived from the Puerto Rican population in PAGE), having too few bands (e.g., two) and permutations per band (e.g., two) results in a relatively higher IBD recovery (94%), but it significantly increases the number of false positives (2259), the runtime (87 s) and memory consumption (6.5 GB). Since the smaller number of hashes is less discriminative, there are many more matches to consider, which consequently increases runtime and memory. A greater number of bands (e.g., 5) and permutations per band (e.g., 4) produces a better tradeoff of 92% accuracy with a minimal number of false positives (14.4), shorter runtime (31.5 s) and smaller memory footprint (5.03 GB) (cf. supplementary Figs. 7 and 8). Having too many minhash values per band results in a slightly lower accuracy, as it makes it harder for segments that are not exact matches to show up among LSH signature hits. A greater number of hashes moderately increases the memory consumption (e.g., 5.5 GB for 6 bands of 6 permutations, cf. Supplementary Fig. 8B), though it does not impact runtime significantly. An increase in the number of bands increases memory consumption and slightly increases runtime, because LSH hashes are stored in a shared hash structure that is created on a single thread and not in a parallelized manner.

Getting a hit among LSH hashes (band hash values) for two slices, does not necessarily mean the two are a match or even similar enough to be considered. iLASH estimates a minimum similarity score based on the number LSH hits between two slices using two thresholds. The *Match Threshold* parameter (MT) controls iLASH decision whether to declare the two slices a match. Slices with estimated similarity scores above the match threshold are queued for extension. If an estimated score is lower than MT, it will be compared to the *Interest Threshold* (IT). Scores that are not declared a match but are higher than interest threshold will be examined on a shingle level in order to find matching sub-slices. iLASH combines neighboring matched slices together to form a longer segment and examines the boundaries of the segment at a shingle level to extend it, if possible. This helps recover as much of the actual IBD segment as possible. For our experiments, we used a match threshold of 99% and interest threshold of 70%.

The iLASH algorithm uses FNV hashing to a 32-bit integer space for both the minhashing and LSH steps. In the LSH step, however, an in-memory hash table was maintained since synchronization is inherently critical for finding hits.

### Pseudocode and complexity

We present the pseudocode of the iLASH algorithm in Box 1 and provide an analysis of its time complexity. ComputeIBD encompasses most of the functionality of the iLASH algorithm. Similar to the description in the methods section, this simplified version of iLASH algorithm first divides the genotype data of the samples (***Haplotypes*)** into a set of slices (***S***) with fixed boundaries on all the samples. Then, for each slice, it tokenizes the genotype data of all the samples, generating the ***TokenSet***. This set is used to make the binary matrix ***D*** with samples as rows and tokens as columns. An element *D*_*i,j*_ for sample *i* and a k-mer value *j* will be assigned a value of one if sample *i* contains the respective k-mer value *j*. Otherwise, it will have a value of zero. Next, we conceptually compute a set of permutations of the columns of the binary matrix ***D***. Each permutation ***π***_***k***_ randomly changes the order of the columns of ***D***, creating a new matrix *π*_*k*_*(****D****)*. The matrix ***H*** is created with rows for samples and columns for permutations. Each element (*H*_*i,k*_) in this matrix corresponds to a sample *i* and permutation *k* pair, and stores the smallest column number on *π*_*k*_*(****D****)* at which the sample *i* has a value of one instead of zero. Next, by banding together the columns of ***H*** in *b* bands for each row and hashing each band to a single value, *b* new hash tables are generated for every slice. Then, for every sample couple *ID*_*1*_*, ID*_*2*_ that share more than *IT* (Interest Threshold) hash values, iLASH creates an entry in the ***MatchSet*** that contains the sample IDs, slice coordinate and the number of hash values shared. The function ExtendSegment then goes through the entries in the ***MatchSet***. For every entry, it first compares the number of shared hash values to *MT* (Match Threshold) and considers entries with equal or higher numbers of shared hash values to be exact matches. Entries that do not pass the threshold will be compared k-mer by k-mer in order for iLASH to find the true boundaries of the matching segment, if any exists. The function will next extend the boundaries of the matches by aggregating neighboring matched slices and k-mer by k-mer comparison of non-matched neighboring slices. The resulting segments are then written to the output file.

We analyze iLASH time and memory complexity in two stages. The first stage comprises reading the genotype data, tokenization, minhashing and generating LSH signatures. The second stage comprises building an integrated hash structure and extraction of matches. Consider a population with $N$ haplotypes, each with a length of $M$ SNPs. Tokenization, minhashing and LSH signature generation each decrease the dimensionality of samples. However, they are applied over the complete dataset. Thus, the time and memory complexity for the first step is $O({MN})$. In the second step, inserting LSH signature values into the hash structure requires $O\left({NV}\right)$, assuming $V$ is the dimensionality of each LSH signature. The number of matched signatures is the crucial factor in the complexity of this stage. The upper bound for number of matches is $\left(\genfrac{}{}{0ex}{}{n}{2}\right)$. However, each population, depending on its structure has a different expected number of matches that can be observed as $\left(\genfrac{}{}{0ex}{}{n}{2}\right)f({p}_{i})$ for population ${p}_{i}$ and $0\le f\left(p\right)\le 1$. Time complexity of iLASH, hence is bounded by $O({N}^{2}f(p)+{MN})$. The same bound also holds true for space complexity, with a different $f{\prime} \left(p\right)$ which addresses the memory growth of the hash table implementation used to store LSH signatures.

Experimentally, we show iLASH’s growth in runtime and memory consumption on the simulated dataset derived from Puerto Rican population in the PAGE study for chromosome 1 in Supplementary Fig. 9. From 1,000 to 80,000 haplotypes, iLASH runtime ranges from 3 to 695 s, and from 437 MB to 49.8 GB. In this range, runtime growth shows a (slightly) quadratic trend, while memory growth is quasi-linear. The quadratic runtime growth is consistent with the growth total length of IBD found by iLASH, which also grows quadratically (cf. Supplementary Fig. 10).

Box 1 iLASH pseudocode **ComputeIBD:**

**INPUT** ***Haplotypes, Band Count*** (***B***), ***Permutations Per Band*** (***R***)

    ***MatchThreshold (MT), InterestThreshold (IT)***

**DEFINE TYPE** *MatchSet* as a tuple with five members (***ID***_**1**_, ***ID***_**2**_***, STARTBP, ENDBP, SIM***)

***sampleCount*** = Number of samples in ***Haplotypes***

*MatchSet* ***Matches***

Split ***Haplotypes*** into a set of contiguous or overlapping slices ***S***

**FOR** every *slice* ***s*** in ***S*** **DO** # *Beginning of the first stage*

    Tokenize all the haplotypes in ***s*** into *k-mers*

    ***TokenSet*** = {Set of all the *k-mer* values that occur in ***s***}

    *Binary Matrix* ***D*** (***sampleCount*** × |***TokenSet***|)

    ***D***_***i,j***_ = 1 if and only if haplotype ***i*** has at least one *k-mer* with

        value equal to ***TokenSet***[***j***]; O otherwise

    P = B*R (total number of permutations)

    ***PermSet*** = {***π*** | ***P*** random permutations of members of ***TokenSet***

       with ***π***(***j***) returning ***TokenSet***[***j***]’s place in the permutation ***π***}

    *Matrix* ***H***(***sampleCount*** × |***PermSet***|)

    ***H***_***i,k***_ = *min*(***π***_***k***_(***j***)| ***D***_***i,j***_ = 1, ***j*** in {1..|***TokenSet***|})

    ***HashTable T***[***B***] # *Beginning of the second stage*

    Divide the columns of ***H*** into ***B*** bands, each encompassing ***R*** values.

    **FOR EACH** ***row*** in ***H*** **DO**

        **FOR EACH** group ***b*** in ***B*** **DO**

            ***T***[***b***] = Hash the ***R*** columns assigned to ***b*** to get a

                 single value.

    **FOR EACH** pair of haplotypes ***ID***_**1**_, ***ID***_**2**_ with at least a common hash

           in ***T DO***

        ***sim*** = Compute similarity between ***ID***_**1**_, ***ID***_**2**_

        **IF** ***sim*** > ***IT*** THEN ***Matches.****add*(***ID***_**1**_, ***ID***_**2**_, ***s.****startbp,* ***s.****endbp*, ***sim***)

**FOR EACH** ***ms*** in ***Matches*DO**

    ***ms*** = *ExtendSegment*(***ms***, ***Haplotypes, MT***)

    **OUTPUT**(***ms***)

***ExtendSegment:***

**INPUT** ***match***, ***Haplotypes, MT***

***ID***_**1**_ = ***match.****ID*_*1*_

***ID***_**2**_ = ***match.****ID*_*2*_

**IF** ***match.****sim* < ***MT DO***

    Scan from ***match.****startbp* to ***match.****endbp* to find the correct start/end of the match by comparing tokens, and    update startbp and/or endbp.

***head*** = ***match.****startbp* − *1*

**WHILE** ***Haplotypes***[***ID***_**1**_,***head***] = ***Haplotypes***[***ID***_**2**_,***head***] ***DO***

    ***head*** = ***head*** − 1

    ***match.****startbp* = ***head***

***tail*** = ***match.****endbp* + *1*

**WHILE*Haplotypes***[***ID***_**1**_,***tail***] = ***Haplotypes***[***ID***_**2**_,***tail***] ***DO***

    ***tail*** = ***tail*** + 1

    ***match.***end*bp* = ***tail***

**RETURN** ***match***

### Other technical notes

To maximize deployment and adoption, iLASH is designed to run on a standard single machine, without requiring parallel technologies like Hadoop or CUDA. However, iLASH takes advantage of the multiple cores available in modern machines and efficiently interleaves computation with reading and writing to disk (I/O operations). To read from and write to files, programs are required to wait in I/O queues. Furthermore, the speed of storage devices is far lower than that of main memory. Therefore, I/O operations can hinder the experiments. iLASH uses parallelization to process the genotypes that are already loaded in memory while it waits for the next batch of data to be loaded. Also, while waiting to write IBD segments to the output file, it computes the next set of IBD segments.

Instead of using the shingles directly, iLASH hashes the shingles using FNV hashing, which has several advantages. First, FNV hashing allows iLASH to deal with different shingle sizes uniformly. It especially helps to compress the shingle space when the shingle length parameter is set to more than 30 bits. Second, FNV hashing enables iLASH to analyze both normalized genotype files (encoded as bit strings of minor/major alleles) and unnormalized files (with for example letter encoding for the bases) in the same fashion. Third, it allows for parallelization, as opposed to using standard C++ hash tables that would create a synchronization bottleneck among the multiple threads. Finally, iLASH also uses FNV to create the permutations to compute minhash signatures. When computing minhash signatures, using FNV to map shingles to a 32-bit space and then using that representation space to create random permutations using a formula instead of actually permuting the matrix, helps maximize the effect of parallelization by eliminating the need to maintain an integrated hash table among all threads. Using x, the hash value of a shingle, as the index number of that shingle, we can generate a new index number for x in a random permutation P(a,b) using the Lehmer random number generator formula^43^; where a and b are randomly selected integers specific to the permutation, and 4294967311 is the largest prime number representable in 32 bits:

1 $${{New\,Index}}_{x}=a\cdot x+{b\,mod}\,4294967311$$

The FNV hash function is also used for generating the LSH signature. However, unlike other steps that involved hashing, analyzing LSH signatures requires maintaining a hash table in order to find hits. Removing in-memory hash structures in shingling and minhashing steps helped us to effectively parallelize our algorithm and gain a substantial speedup against our original implementation.

### Test data generation

We used HAPGEN2 to simulate our large genotype dataset (with the following command-line arguments: *-Ne 80000 -theta 130*). We chose a high mutation rate in order to minimize background IBD.

To simulate genotype data of individuals for the false-positive rate experiments, we took a composite individual approach^23^. Genotype data of African American individuals in the PAGE Study was broken down in short windows and randomly rearranged to eliminate IBD while preserving LD structure. We then randomly copied haplotypes between different individuals to simulate IBD for power tests. The number and the length of these haplotypes was sampled from the PAGE study IBD estimation results.

### Application to population architecture using genomics and epidemiology (page) study data

A total of 51,520 subjects were genotyped on the MEGA array as part of the Population Architecture using Genomics and Epidemiology (PAGE) study^30^. Genotypes that passed quality control (*N* = 1,402,653) underwent phasing using SHAPEIT2. At this stage an additional *N* = 1 individual was removed for having a chromosome specific missingess rate of >10%. Phased haplotypes for the autosomes were subsequently filtered to a minor allele frequency of 5% and indels were removed (resulting in the retention of 593,326 autosomal SNPs genome-wide). SHAPEIT2 output was then converted to plink format using the fcgene software and these files were used as the input for both GERMLINE and iLASH (along with genetics maps interpolated using data from b37 genetic maps). GERMLINE is known to have a high rate of false-positive for short segments (<4 cM) when used without the “-haploid” flag [cite https://pubmed.ncbi.nlm.nih.gov/28176766/]. In the experiments for this paper, we use phased haplotype data exclusively. Thus, we always passed the “-haploid” flag, which prevents the false-positives issues on GERMLINE.

To compute IBD we used comparable parameters for GERMLINE and iLASH. The flags used for GERMLINE were *“-min_m 3 -err_hom 0 -err_het 2 -bits 25 –haploid.”* For iLASH the parameters were: “*auto_slice 1*, *shingle_size 20*, *shingle_overlap 0*, *bucket_count 5, perm_count 20, max_thread 20, match_threshold 0.99, interest_threshold 0.70*, *max_error 0*, *min_length 3, cm_overlap 1.4.”*

The code with detailed descriptions iLASH’s input parameters, along with a discussion of best practices and other recommendations, is available in the online user manual at https://github.com/roohy/iLASH.

The scripts used for simulations are also available at https://github.com/roohy/ilash_analyzer.

### Quality control for downstream analysis in PAGE

IBD haplotypes inferred for *N* = 38,919 PAGE individuals from the WHI, MEC, and HCHS/SOL studies were filtered for regions that overlapped with known genomic regions of low complexity. In addition, IBD haplotypes that fell within genomic regions of excess IBD sharing (empirically defined as regions where we observed that the mean number of inferred IBD haplotypes exceeded 3 standard deviations of the genome-wide mean) were also excluded from downstream analysis.

### Identity-by-descent network construction and community detection

The length of IBD haplotypes (cM) shared between each pairs of PAGE individuals were summed to obtain the total length of IBD shared genome-wide between pairs. This was used as the input for the construction of an undirected network using the iGraph software in R (version 3.2.0) where each individual was represented as a node, weighted edges were used to represent the sum of IBD sharing between any given pair. Community detection was then performed using the infomap.community() function from the iGraph package.

### Application to UK Biobank data

IBD calling on the UK Biobank was performed on phased haplotype data^44^. Phased haplotype data for *N* = 487,330 UK Biobank participants in BGEN v1.2 format were converted to vcf format using bgenix (v1.0.1) and subsequently converted to PLINK format using an in-house python script. After the removal of indels, a total of 655,532 SNPs were retained across the autosomes. These sites were used as the input for iLASH, which was run using the following parameters:

*“perm_count 12, shingle_size 20, shingle_overlap 0, bucket count 4, max_thread 20, match_threshold 0.99, intersect_threshold 0.70, max_error 0, min_length 2.9, auto_slice 1, cm_overlap 1.4*”

## Supplementary information

Supplementary Information

## Data Availability

The Population Architecture using Genomics and Epidemiology (PAGE) data is available through dbgap at accession phs000356. [https://www.ncbi.nlm.nih.gov/projects/gap/cgibin/study.cgi?study_id=phs000356.v2.p1]. The UK Biobank data is available through its website [https://www.ukbiobank.ac.uk/enable-your-research/about-our-data] via application. Source data are provided with this paper. We plan to contribute the IBD segments for UK Biobank to the UK Biobank Portal. Furthermore, we have provided the required parameters to generate IBD segments in the PAGE study dataset using iLASH in the Methods section.

## Source data

Source Data

## Code availability

The iLASH code, manual, description of best practices, and additional scripts are available at https://github.com/roohy/iLASH (10.5281/zenodo.4433075).

The scripts used for the simulations are also available at https://github.com/roohy/ilash_analyzer (10.5281/zenodo.4433081).

iLASH is available under the USC Software License, which allows free use for academic and non-commercial purposes.
