# Supplementary material for: Rapid detection of identity-by-descent tracts for mega-scale datasets
Source: Nat Commun. 2021 Jun 10;12:3546. doi: 10.1038/s41467-021-22910-w (PMC8192555; doi:10.1038/s41467-021-22910-w)
Supplement: Supplementary file 1 — Supplementary Information [file 41467_2021_22910_MOESM1_ESM.pdf]

Supplementary Information for: R Shemirani, GM Belbin, CL Avery, EE Kenny, CR Gignoux, and JL Ambite: "Rapid detection of identity-by-descent tracts for mega-scale datasets"

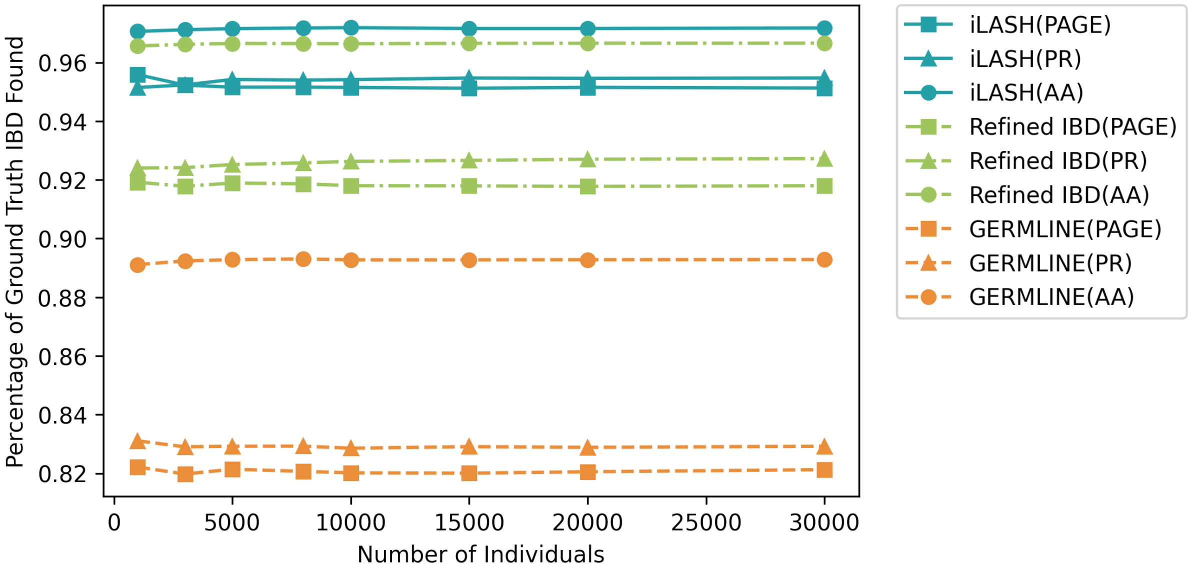

Supplementary Figure 1. Concordance of results with the ground truth. Concordance of iLASH, Refined IBD, and GERMLINE with ground truth on simulated data from three populations based on distributions found on the PAGE study: African Americans (AA), Puerto Ricans (PR), and all subjects (PAGE). Source data are provided as a Source Data file.

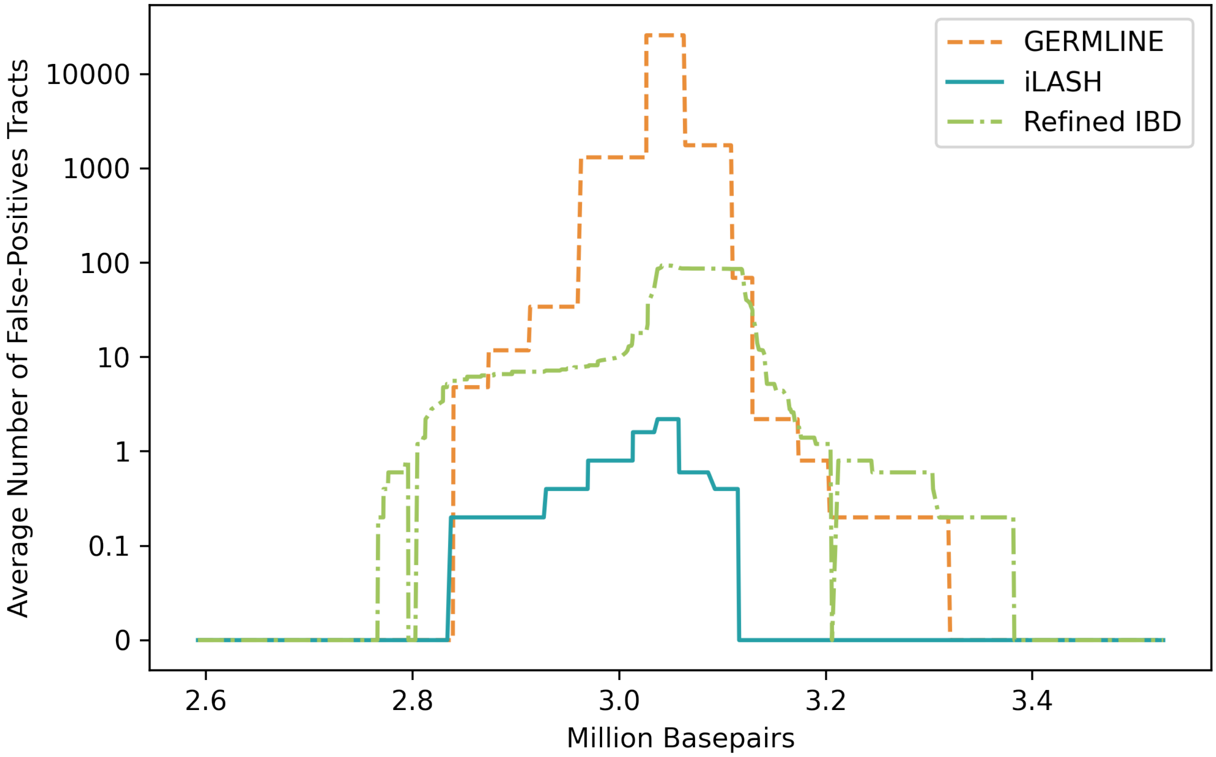

Supplementary Figure 2. Number of false-positive segments recovered. A logarithmic scale heatmap of the average number of false-positive tracts estimated by iLASH, GERMLINE, and Refined IBD on chromosome 2 between base pairs 2766642 and 3042093 using composite individual approach to generate 2,000 unrelated samples. To calculate the average values, the experiment was repeated 5 times with new simulated data. Source data are provided as a Source Data file.

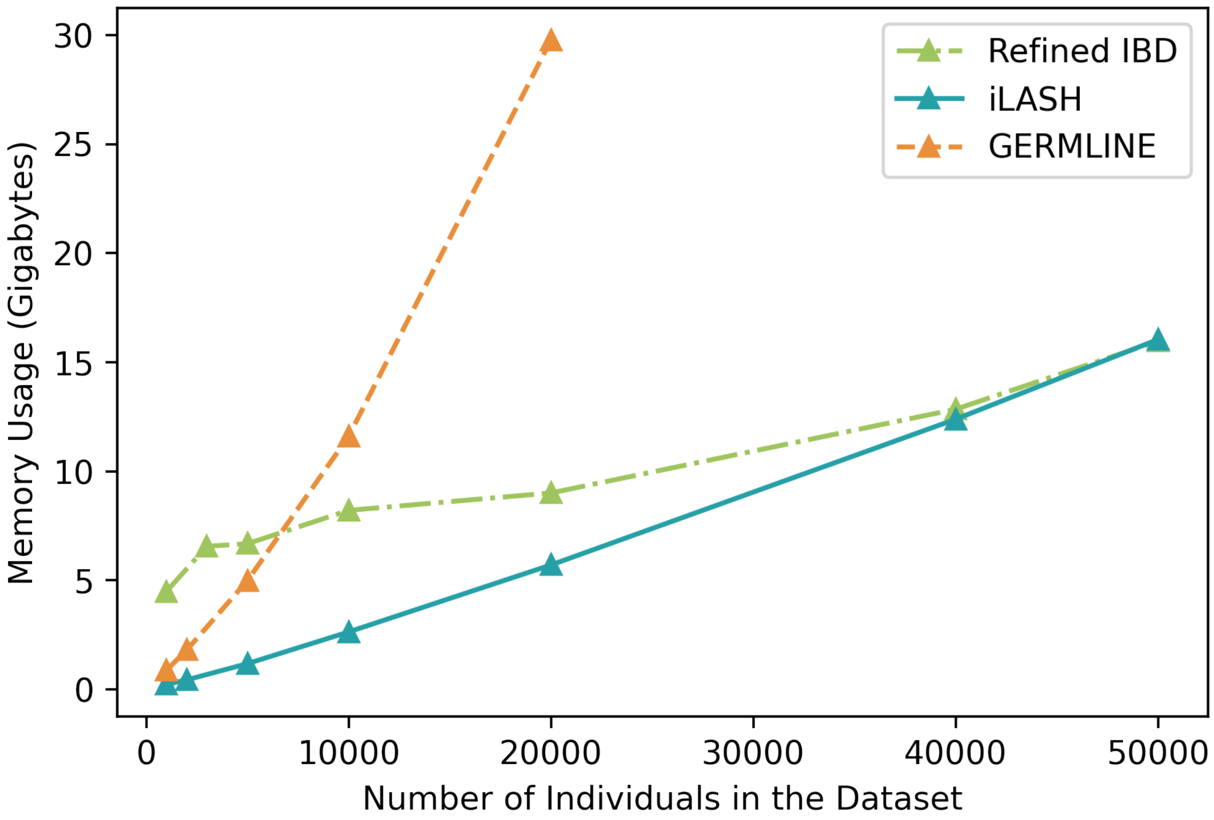

Supplementary Figure 3. A comparison of memory usage of algorithms. iLASH, Refined IBD, and GERMLINE memory usage trends as the number of samples grows using the simulated data derived from the Puerto Rican population in the PAGE study on chromosome 1. Source data are provided as a Source Data file.

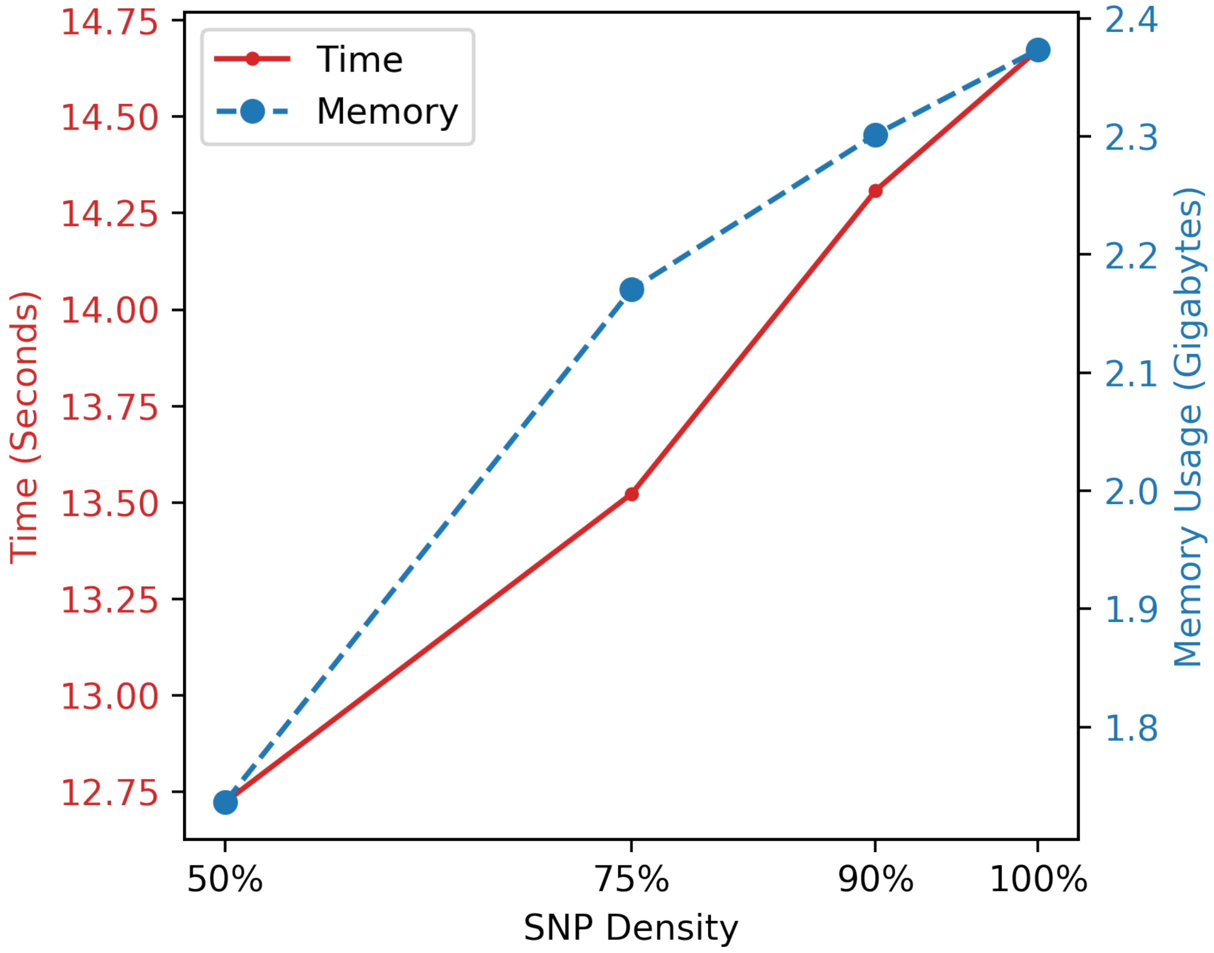

Supplementary Figure 4. Runtime and memory usage trends based on array density. Average iLASH runtime and memory consumption growth based on SNP density of the genotype data. Based on the simulated data (5000 samples) derived from Puerto Rican population on PAGE study on Chromosome 1 with 116,415 SNPs at 100%, averaged over 10 runs, each having using a new simulation. Source data are provided as a Source Data file.

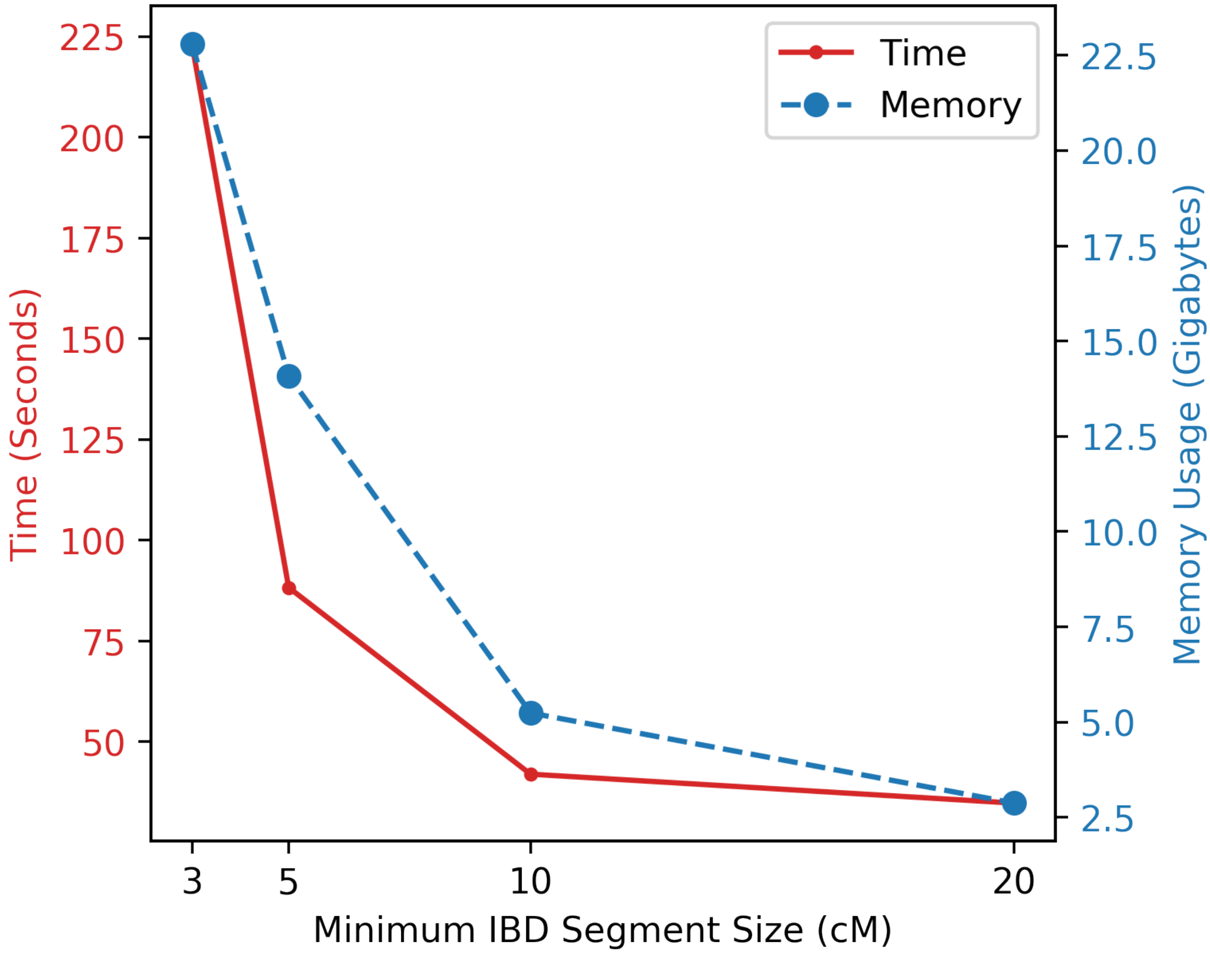

Supplementary Figure 5. Runtime and memory usage trends based on minimum segment size. The effect of minimum IBD segment size (in cM) on the average runtime and memory usage of iLASH. Based on our simulated data on Chromosome 1 for 40,000 samples, averaged over 10 runs. Source data are provided as a Source Data file.

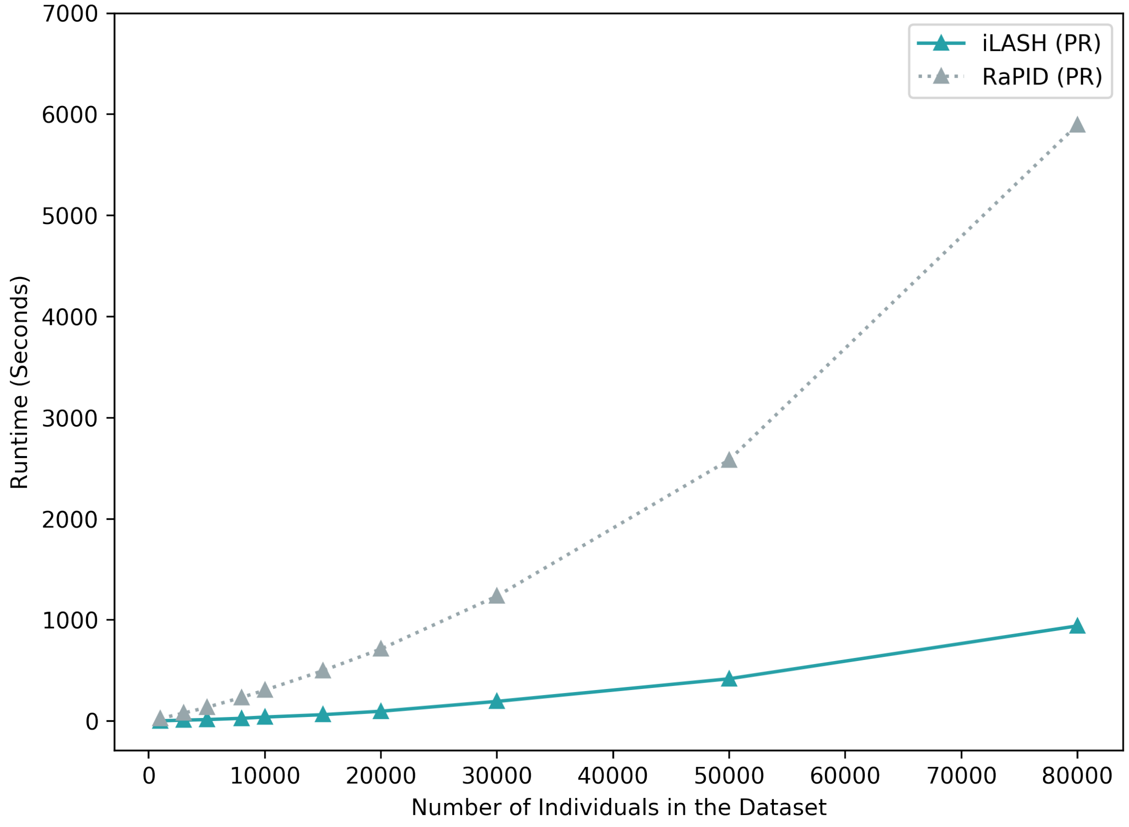

Supplementary Figure 6. Runtime comparison of iLASH and RaPID. iLASH-RaPID runtime comparison based on dataset size using simulated data derived from the Puerto Rican (PR) population in the PAGE study. Source data are provided as a Source Data file.

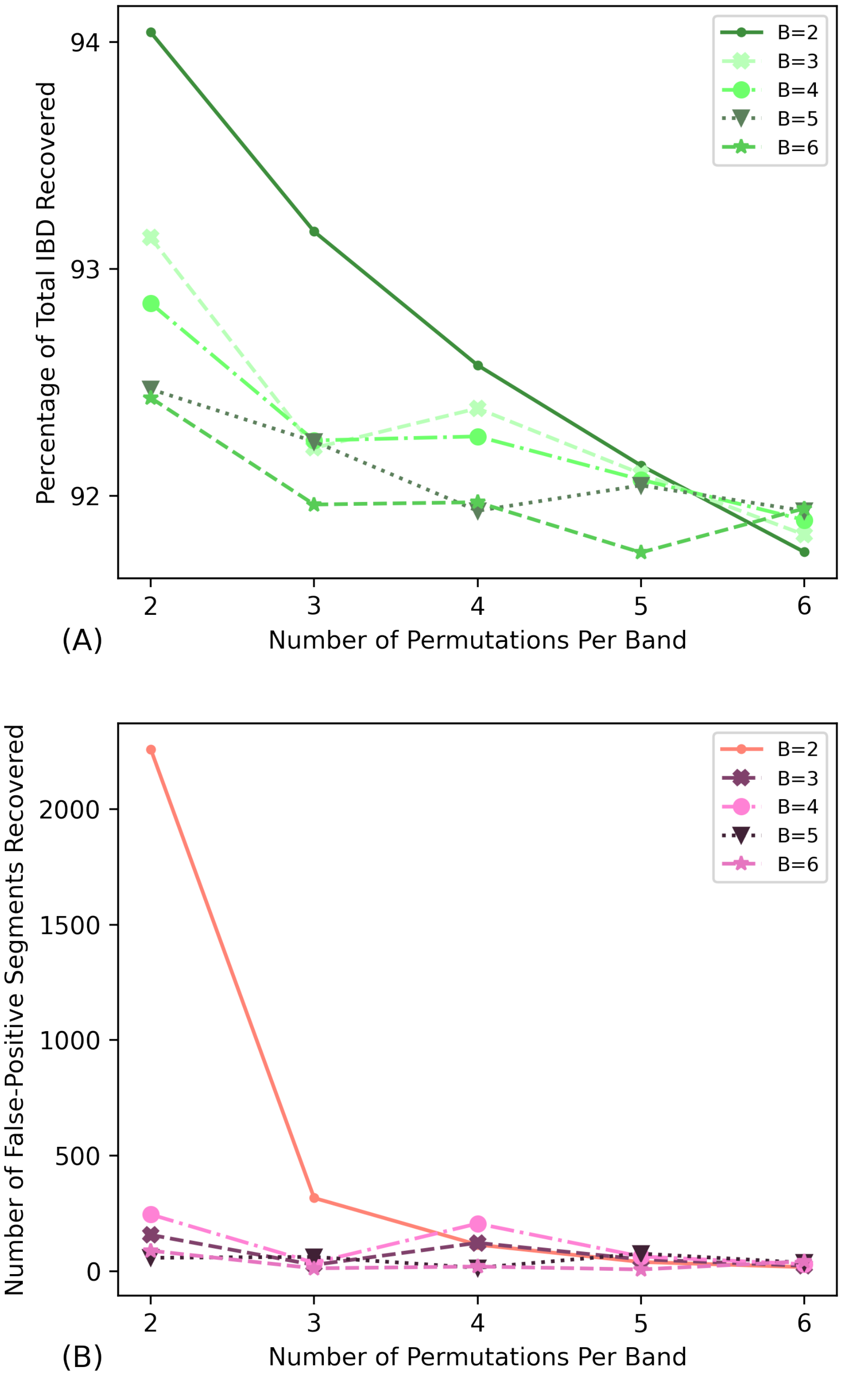

Supplementary Figure 7. Effects of LSH parameters on accuracy and false-positive rates. Effects of different band counts *B* and permutations per band on the (A) average accuracy on simulated data with 10,000 samples derived from Puerto Rican population in the PAGE study, repeated 10 times; and on the (B) average number of false-positive recovered, using composite individual dataset of 2,000 samples on chromosome 2, averaged over 10 runs. Source data are provided as a Source Data file.

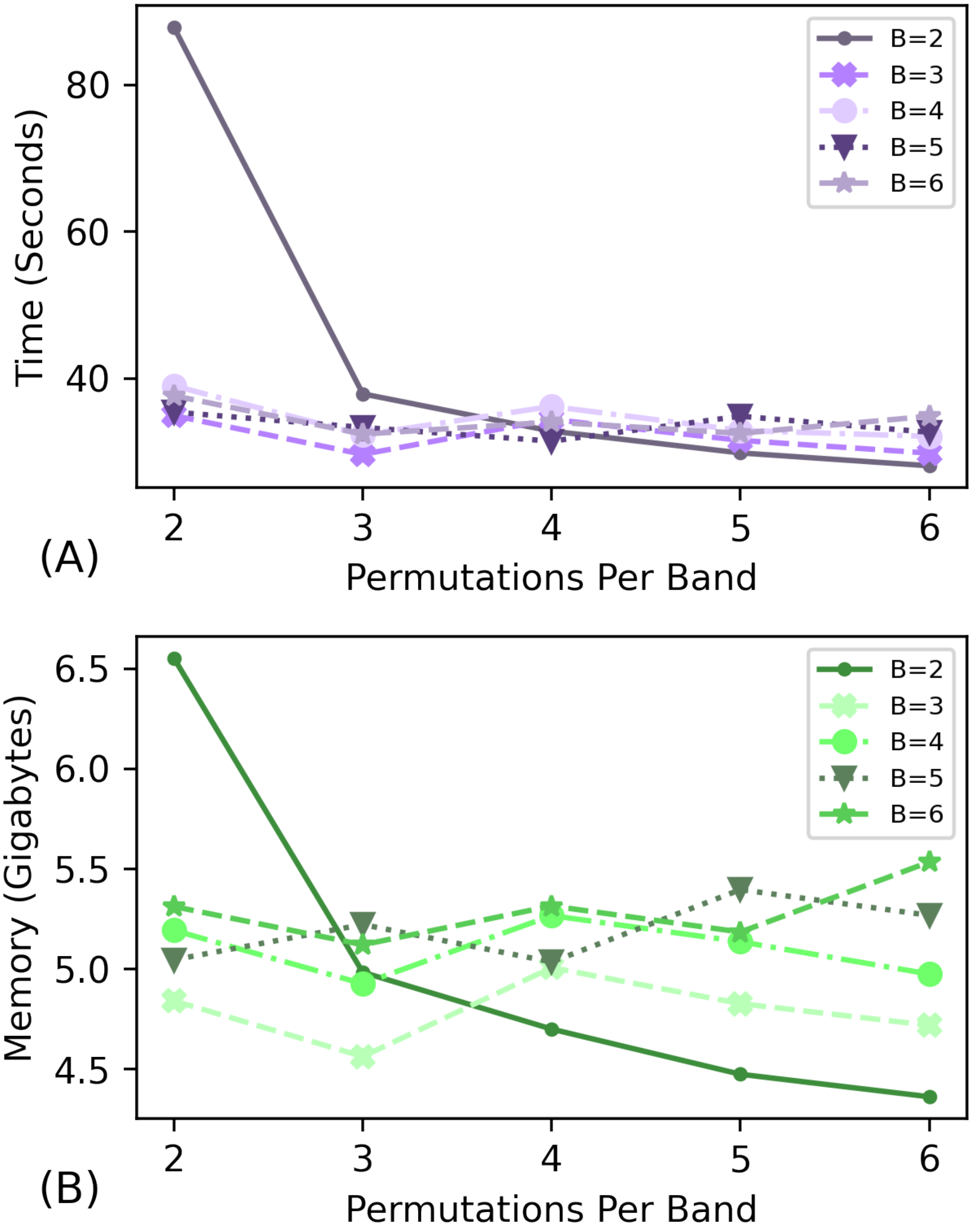

Supplementary Figure 8. Effects of LSH parameters on Memory usage and runtime of iLASH. Effects of different band counts *B* and permutations per band on (A) time, and (B) on memory usage of iLASH while running on simulated data with 10,000 samples derived from Puerto Rican population in the PAGE study, repeated 10 times. Source data are provided as a Source Data file.

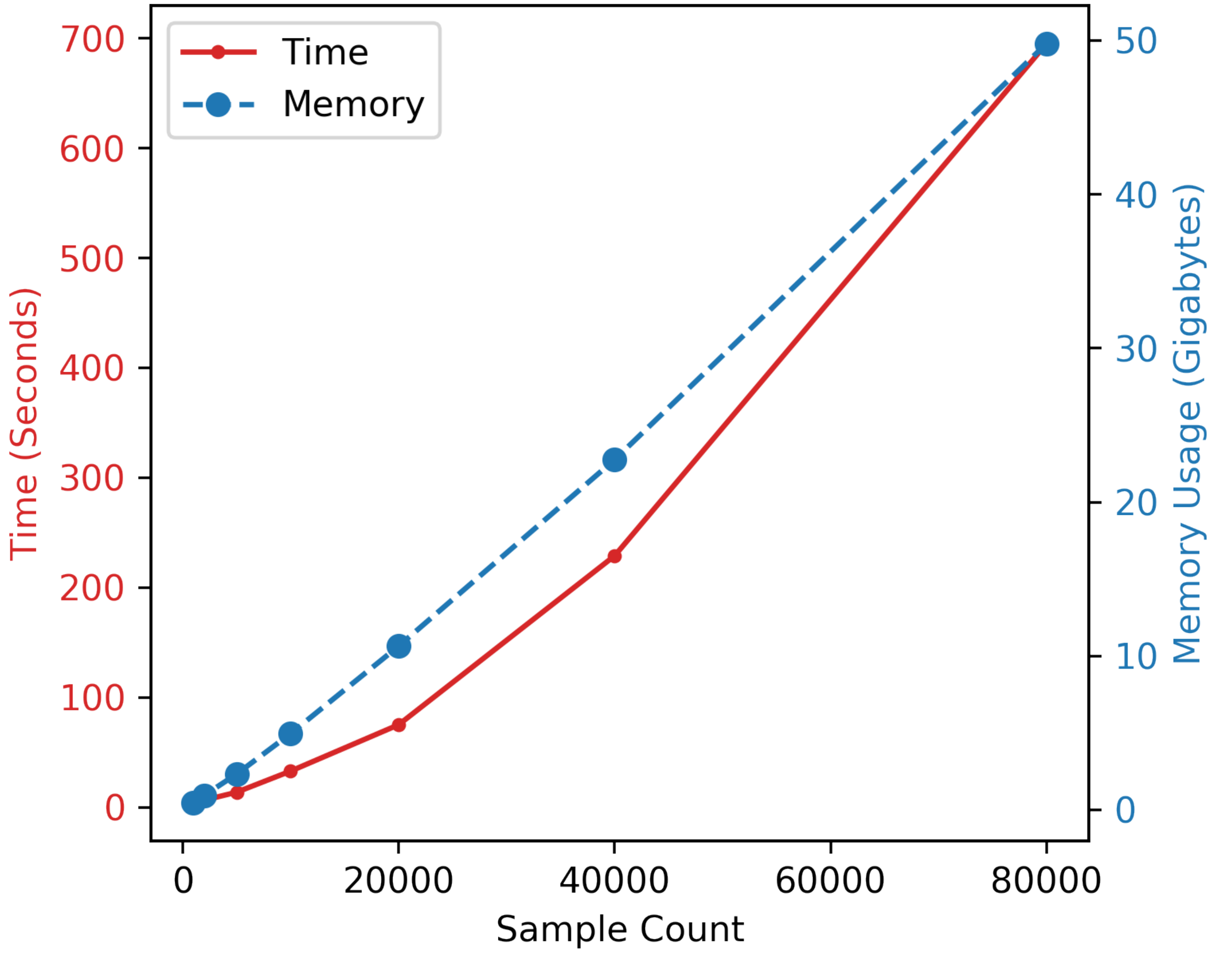

Supplementary Figure 9. Runtime and memory usage trends based on the number of samples. Time and memory growth of iLASH compared to the sample sizes. Using our simulated dataset derived from Puerto Rican population in the PAGE study (chromosome 1). Source data are provided as a Source Data file.

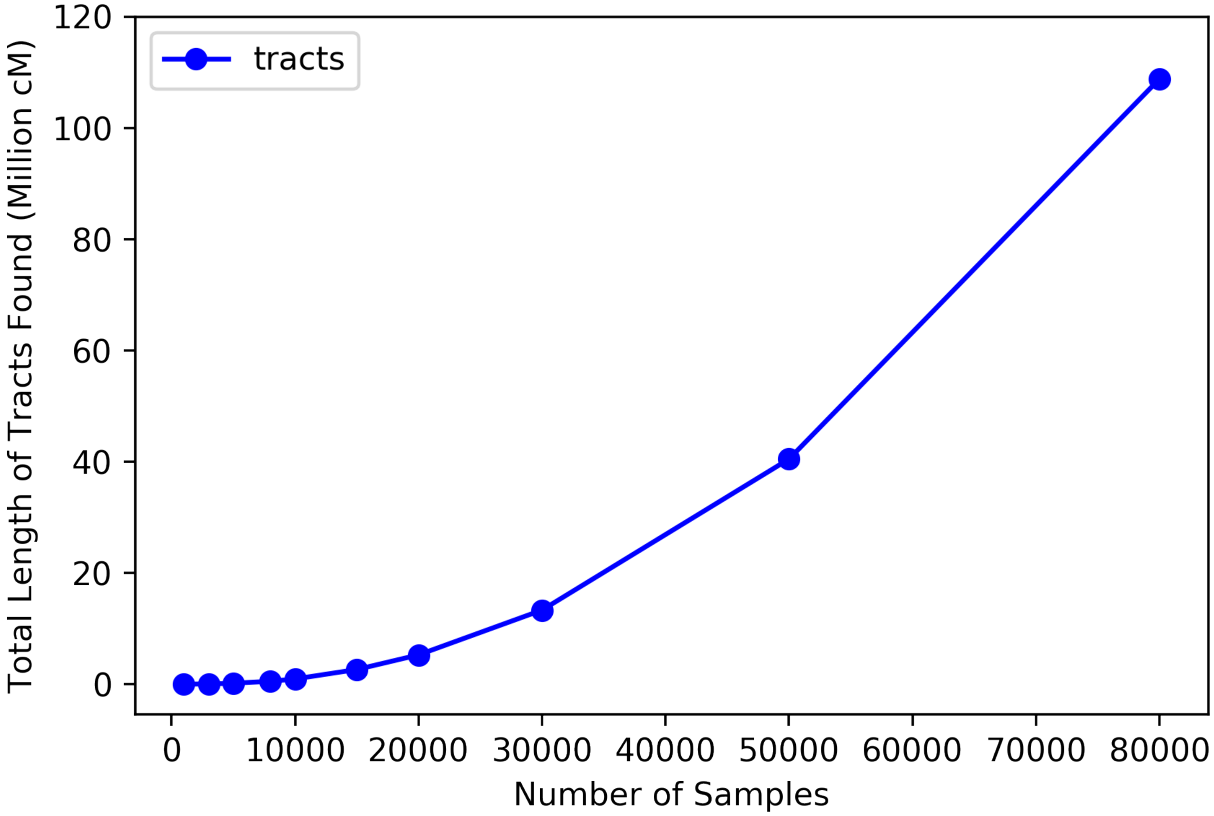

Supplementary Figure 10. Total length of simulated IBD as the number of samples increases. Growth in the total length of simulated IBD compared to the number of samples in our simulated datasets derived from Puerto Rican population in the PAGE study (chromosome 1). Source data are provided as a Source Data file.

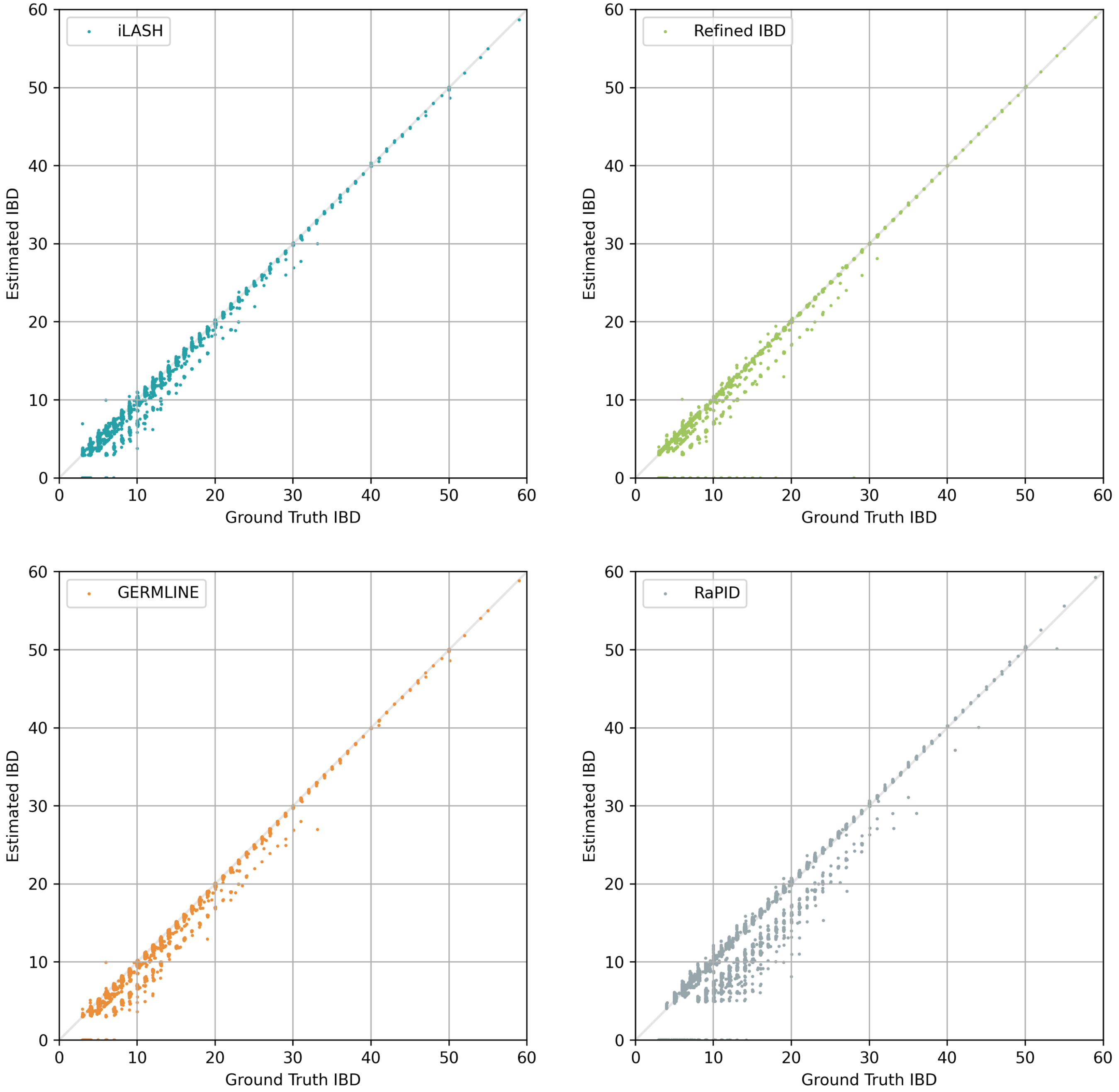

Supplementary Figure 11. Segment-wise comparison of ground truth IBD length versus estimated IBD length. A scatterplot of total estimated IBD by iLASH, Refined IBD, GERMLINE, and RaPID against the ground truth IBD between haplotypes in a simulated dataset of 2,000 people derived from Puerto Rican population in PAGE (Chromosome 1). 196,913 simulated IBD segments are present in this dataset. Background IBD is removed through "composite individuals" method. Source data are provided as a Source Data file.

Supplementary Table 1.

Community	PPV	Top Label
1	0.954943274	AfricanAmerican
2	0.985674085	NativeHawaiian
3	0.958929226	Mexico
4	0.955396256	PuertoRico
5	0.975548061	Japan
6	0.978395062	Cuba
7	0.980787704	DominicanRepublic
8	0.428571429	Mexico
9	0.996835443	Mexico
10	0.972972973	Puno
11	0.5	PuertoRico
14	0.571428571	Ecuador
